# Selective inhibition of human translation termination by a drug-like compound

**DOI:** 10.1038/s41467-020-18765-2

**Published:** 2020-10-02

**Authors:** Wenfei Li, Stacey Tsai-Lan Chang, Fred. R. Ward, Jamie H. D. Cate

**Affiliations:** 1 Department of Molecular & Cell Biology, University of California, Berkeley, CA 94720 USA; 2 Innovative Genomics Institute, University of California, Berkeley, CA 94720 USA; 3 Molecular Biophysics and Bioimaging Division, Lawrence Berkeley National Laboratory, Berkeley, CA 94720 USA; 4 Department of Chemistry, University of California, Berkeley, CA USA

**Keywords:** Mechanism of action, Ribosome, Electron microscopy

## Abstract

Methods to directly inhibit gene expression using small molecules hold promise for the development of new therapeutics targeting proteins that have evaded previous attempts at drug discovery. Among these, small molecules including the drug-like compound PF-06446846 (PF846) selectively inhibit the synthesis of specific proteins, by stalling translation elongation. These molecules also inhibit translation termination by an unknown mechanism. Using cryo-electron microscopy (cryo-EM) and biochemical approaches, we show that PF846 inhibits translation termination by arresting the nascent chain (NC) in the ribosome exit tunnel. The arrested NC adopts a compact α-helical conformation that induces 28 S rRNA nucleotide rearrangements that suppress the peptidyl transferase center (PTC) catalytic activity stimulated by eukaryotic release factor 1 (eRF1). These data support a mechanism of action for a small molecule targeting translation that suppresses peptidyl-tRNA hydrolysis promoted by eRF1, revealing principles of eukaryotic translation termination and laying the foundation for new therapeutic strategies.

## Abstract (web summary)

The drug-like compound PF846 and its derivatives inhibit the translation of specific mRNAs by the human ribosome. Here the authors show how PF846 arrests translation at the stop codon by slowing hydrolysis of the protein nascent chain at the ribosome P-site tRNA by eukaryotic release factor 1 (eRF1).

## Introduction

Many diseases are affected by proteins that have been difficult or impossible to target directly with small-molecule therapeutics. To treat these diseases, efforts to expand the druggable proteome have attempted to harness knowledge of endogenous cellular pathways to alter gene expression. For example, compounds termed PROteolysis TArgeting Chimeras (PROTACs) serve as molecular recruiters that can direct specific proteins to the ubiquitin–proteasome protein degradation machinery^1^. Other strategies use small molecules that bind messenger RNAs (mRNAs) to inhibit their translation, or lead to RNA degradation by cellular quality control systems^2^. Recently, small molecules have even been developed to direct secreted proteins to the lysosome for degradation^3^. However, ensuring the selectivity of these new small-molecule-based strategies is complicated by the fact that they tap into endogenous regulatory pathways with a large number of physiological substrates, requiring a deep understanding of the molecular basis for targeting specificity.

Recently, we described the drug-like compound PF846 and its derivatives that target proteins by inhibiting the translation of specific mRNAs by the human ribosome^4–7^. These small molecules bind in the ribosome exit tunnel in a eukaryotic-specific pocket formed by highly conserved 28S ribosomal RNA (rRNA) residues^4,6,7^. Remarkably, they are able to inhibit protein synthesis in a highly selective manner dependent on the nascent polypeptide sequence, opening fundamentally new ways to target proteins of therapeutic interest^4,7^. Initially discovered to stall translation elongation^4^, PF846 prevents the movement of mRNA and tRNA on the ribosome by disrupting proper binding of the peptidyl-tRNA in the ribosome peptidyl transferase center^4,7^.

Surprisingly, PF846 can also block translation termination of specific polypeptides^7^. Translation termination occurs when an mRNA stop codon enters the ribosome A site. In eukaryotes, termination is mediated by eRF1 and eRF3 (eukaryotic release factors (RFs) 1 and 3), which form a ternary complex with GTP^8^. Following stop codon recognition by the eRF1 N-terminal domain, eRF1 rearranges into an extended state that inserts a GGQ motif (Gly–Gly–Gln) into the PTC to release the nascent peptide^9–11^. After the nascent peptide is released, the ATPase ABCE1 promotes eRF1 dissociation from the ribosome and ribosome recycling^12,13^. Differences in the mechanisms of translation elongation and termination suggest that PF846 may act in a different manner in these two steps.

In this work, we use a combination of cell-based, cryo-EM, and biochemical studies to decipher how the drug-like compound PF846 inhibits translation termination on human ribosomes. We show that PF846 stalls translation termination both in an in vitro translation system and in cells in a sequence-dependent manner. We also present two structures of PF846-stalled ribosome-NC (RNC) complexes, along with biochemical experiments, that reveal the steps before and during translation termination that are inhibited by the compound. Taken together, our data support a model in which PF846 inhibits translation termination by first slowing down translation elongation before reaching the stop codon and subsequently blocking NC hydrolysis from the P-site tRNA, thereby trapping the NC on the ribosome.

## Results

### Selective inhibition of translation termination by PF846 in cells

**Fig. 1:**
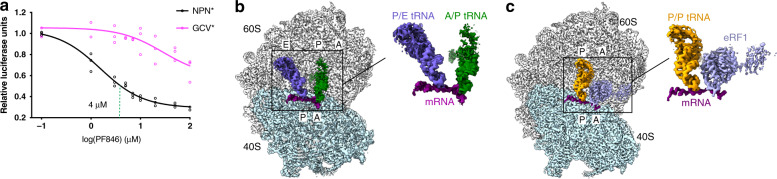
Cell-based assays and structural analysis of PF846-stalled termination complexes. **a** Luciferase reporter assays of CDH1–NPN* (black circles) and CDH1–GCV* (magenta circles) stable cell lines in response to PF846 with different concentrations of 0, 1, 3, 5, 7, 10, 20, 50, and 100 μM (data present mean ± s.d., *n* = 3 independent experiments). The vertical line indicates the PF846 concentration when the fitted line crosses 0.5. From the CDH1–GCV* data, the IC_50_ of nonspecific inhibition is at least 100 μM PF846. **b** Structure of CDH1–NPN* RNC in the rotated state bearing A/P-site (dark green) and P/E-site tRNAs (slate blue), with mRNA (magenta), the 40S subunit (light cyan) and 60S subunit (gray). A close-up view of the mRNA and tRNAs is shown to the right. **c** Cryo-EM reconstruction of CDH1–NPN* RNC in the nonrotated state, with eRF1 (slate blue), and P/P-site tRNA (orange). A close-up view of the mRNA, tRNA, and eRF1 is shown to the right.

Earlier, we had shown using an mRNA library-based approach that PF846 can stall translation termination of specific protein NC sequences^7^. In the library, which encoded a segment of human CDH1 (Cadherin-1) previously shown to stall translation elongation, we randomized four amino acids near the PTC and identified a strong enrichment of sequences with a stop codon (UAG)^7^. In vitro translation assays showed that PF846 prevented NC release from the ribosome when CDH1 sequences contained Asn–Pro–Asn (NPN) but not Gly–Cys–Val (GCV) preceding the stop codon^7^. Using the CDH1 sequence ending with NPN (CDH1–NPN*, with * being the stop codon), we first tested whether PF846 can selectively inhibit translation termination in cells. We engineered a stable human cell line (HEK293T) expressing a nanoluciferase reporter fused at its C terminus to the CDH1–NPN polypeptide (Supplementary Fig. 1a), along with a second cell line replacing the NPN* motif with GCV* (CDH1–GCV*), which is not responsive to PF846 in vitro^7^. Treatment of cells with PF846 showed that the compound inhibits the expression of nanoluciferase fused to CDH1–NPN*, with an IC_50_ around 4 µM, but only weakly inhibits expression of nanoluciferase fused to CDH1–GCV* at high doses of PF846 (Fig. 1a, Supplementary Fig. 1b) consistent with in vitro translation results^7^.

### Cryo-EM analysis of a PF846-stalled translation termination complex

**Fig. 2:**
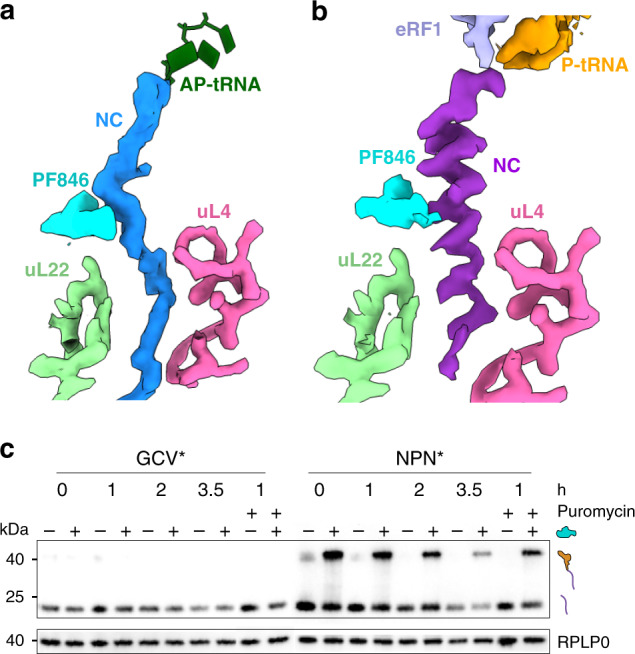
Overview of PF846-stalled nascent chains in the ribosome exit tunnel. **a** The PF846-stalled nascent chain from the rotated RNC (light blue). Surface representations of the segmented cryo-EM density for PF846 and ribosomal proteins are indicated. **b** Cryo-EM density for the stalled nascent chain within the nonrotated RNC (purple). Surface representations of the segmented cryo-EM density for eRF1, P-site tRNA, PF846, and ribosomal proteins are indicated. **c** Western blots of CDH1–GCV* and CDH1–NPN* RNCs in the presence (+) or absence (−) of 50 μM PF846 on different time scales at room temperature. Puromycin treatment was performed on pelleted RNCs at room temperature for 1 h with a final concentration of 1 mM puromycin. The experiment shown in **e** was repeated three times independently with similar results.

To determine the structural basis for how the small-molecule PF846 inhibits translation termination, we isolated human RNC complexes from in vitro translation reactions programmed with an mRNA encoding the CDH1–NPN* fusion protein (Supplementary Fig. 2a). We then used cryo-EM to determine structures of the PF846-stalled termination complexes. Particle sorting of the cryo-EM data yielded a minor population of the RNCs comprising about one-third of the particles in the rotated state, bearing tRNAs in the hybrid aminoacyl site/P site (A/P site) and P site/exit site (P/E site) (Fig. 1b, Fig. 2a, Supplementary Fig. 2b). The conformation of the RNC complex in the rotated state, including the NC, is similar to that of PF846-stalled RNCs during translation elongation (Fig. 1b, Fig. 2a)^4,7^. Also as previously seen with stalled elongation complexes, including the original CDH1 stalling sequence, the NC density could not be resolved at the amino acid level, as it likely consists of different sequences superimposed on each other^4,7^. This is supported by the blurred cryo-EM density for the mRNA codon–tRNA anticodon base pairs in the mRNA decoding center (Supplementary Fig. 3a–c), as observed in PF846-stalled translation elongation complexes^4,7^. Taken together, these structural features of the minor population are consistent with PF846 slowing translation of the CDH1–NPN polypeptide prior to reaching the stop codon.

**Fig. 3:**
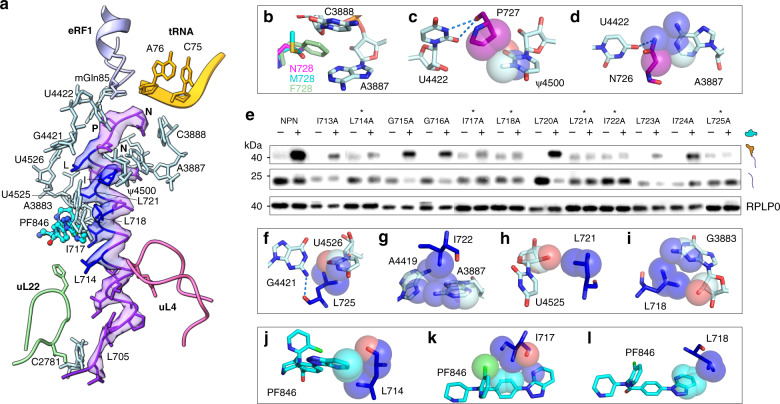
Structural details of the nascent chain in the nonrotated state and effects of mutations in the NC. **a** The CDH1–NPN nascent-chain model within the cryo-EM density (transparent surface). rRNA bases (light cyan) and ribosomal protein residues (light green for uL22 and pink for uL4) that have close interactions with the nascent chain are shown. The amino acids of the nascent chain found to be essential for PF846-mediated stalling are colored blue. Amino acids without numbers are I722, L725, N726, P727, and N728 at the C terminus. **b** Models of different amino acids at the NC C-terminal position 728. The pocket formed by 28S rRNA nucleotides A3887 and C3888 can accommodate different sizes of amino acid, with methionine (M728) and phenylalanine (F728) as examples. **c**, **d** Interactions between P727 and N726 with adjacent 28S rRNA nucleotides. Dashed lines indicate hydrogen bonds and spheres represent van der Waals radii of C, N, and O atoms. **e** Western blots of FLAG-tagged CDH1–NPN nascent chains containing single mutations, from in vitro translation reactions in the presence (+) or absence (−) of 50 μM PF846. The mutations that affect termination are indicated with asterisks. The positions of tRNA-bound and free nascent chains are shown, with RPLP0 serving as a loading control. **f**, **i** Interactions between 28S rRNA nucleotides and the PF846-stalled nascent chain in the nonrotated RNC with dashed lines indicating hydrogen bonds and spheres representing van der Waals radii. **j**–**l** Direct interactions between PF846 and nascent-chain residues. Spheres represent van der Waals radii. O, N, and Cl atoms are colored in red, blue, and green, respectively. The experiment shown in **e** was repeated three times independently with similar results.

Interestingly, the major population of ribosomes in the cryo-EM data adopts an entirely different conformation, the nonrotated state, with an overall resolution of 2.8 Å (Fig. 1c, Supplementary Figs. 2b, 3d–f, 4, and 5) sufficient to visualize chemical modifications of rRNA and tRNA nucleotides (Supplementary Fig. 6)^14–16^. In the resulting maps of this state, the small-molecule PF846 is also well resolved, along with the protein NC, P-site tRNA, and eRF1 (Fig. 1c, Fig. 2b, Supplementary Fig. 5)^17^. The global conformation of the ribosome resembles that observed previously for eRF1-bound translation termination complexes^18,19^, consistent with the structure representing a stalled termination complex. In the structure, the protein NC is well defined in the ribosome exit tunnel with an average resolution of 3–3.5 Å, allowing us to accurately model the NC conformation and sequence register (Fig. 2b, Supplementary Fig. 5c). In striking contrast to the NC in PF846-stalled translation elongation complexes, the NC in the stalled termination complex adopts an α-helical geometry spanning 21 amino acids (residues 705–725), followed by the NPN motif (residues 726–728) in an extended conformation (Fig. 2 and Fig. 3).

In the PF846-stalled termination complex, the cryo-EM density for the ester bond between the NC and P-site tRNA is weak (Supplementary Fig. 7a, b), suggesting that some of the NC has been released from the P-site tRNA, but remains trapped in the ribosome exit tunnel. We therefore tested RNC formation in vitro to assess the status of the isolated NCs. In samples affinity-purified in the same manner as for cryo-EM analysis, which includes vigorous washing steps at room temperature, both NC–tRNA and NC released from P-site tRNA are present in the RNCs (Supplementary Fig. 7c). Notably, in in vitro translation reactions, CDH1–NPN* NC–tRNA hydrolysis is significantly slowed in a PF846-dependent and NC sequence-dependent manner (Fig. 2c and Supplementary Fig. 8). However, NC released from P-site tRNA remains bound to ribosomes, even in the absence of PF846 (Fig. 2c and Supplementary Fig. 8b). This is also true for CDH1–GCV* but not for the nanoluciferase NC (Fig. 2c and Supplementary Fig. 8b). These results indicate that the cryo-EM density likely reflects a mixture of NC–tRNA and NC hydrolyzed from P-site tRNA, although the released NC is trapped in the ribosome exit tunnel independent of PF846.

### Interactions of the NC with the ribosome exit tunnel and PF846

The unexpected ability of PF846 to trap the NC in the ribosome exit tunnel at the stop codon prior to NC–tRNA hydrolysis (Fig. 1c, Fig. 2, Supplementary Fig. 7, Supplementary Fig. 8) led us to investigate the contribution of single amino acid residues of the NC during PF846-induced stalling. We previously identified the NPN motif (residues 726–728) in an mRNA library-based experiment, in which a range of sequences in these positions supported inhibition of translation termination^7^. Within the library, the amino acid in position 728 could accommodate nearly all other amino acids, except for the largest two, tyrosine and tryptophan (Y and W)^7^. In support of these findings, N728 projects toward a pocket formed by 28S rRNA residues G3886–C3888 that leaves room for larger amino acid side chains (Fig. 3a, b and Supplementary Fig. 9). P727 makes multiple contacts to U4422 and Ψ4500 in 28S rRNA (Fig. 3c), and cannot tolerate larger amino acids, again consistent with enrichment of predominantly P and V at this position^7^. N726 makes hydrogen bond and van der Waals contacts to U4422 and A3887, and can tolerate replacement with amino acids D and H, as seen in the previously identified sequence motif (Fig. 3d)^7^.

To test whether the NPN sequence is sufficient for PF846-dependent inhibition of translation termination, we examined the NPN motif in the context of PCSK9 (Proprotein convertase subtilisin/kexin type 9). Notably, the PCSK9 sequence originally identified as sufficient for PF846 to stall translation elongation^4,7^ is not predicted to form a compact α-helical geometry as observed in the CDH1–NPN* structure (Supplementary Fig. 8a). We inserted the NPN* motif at different positions in the PCSK9 sequence (Supplementary Fig. 8c). In contrast to the effects of PF846 on PCSK9 during translation elongation, PF846 was unable to potently inhibit translation of any of the C-terminal PCSK9–NPN* sequences (Supplementary Fig. 8b, c). Interestingly, although the PCSK9–NPN nascent chains are not sensitive to PF846, they can remain associated with RNCs after NC release from P-site tRNA (Supplementary Fig. 8b), as observed for CDH1–GCV*.

Consistent with the observation above that the NPN motif is necessary but not sufficient for PF846-dependent inhibition of translation termination, mutation of L725 in the CDH1–NPN NC abolishes translational stalling (Fig. 3e and Supplementary Fig. 10g). In the structure of the stalled termination complex, L725 makes a backbone hydrogen bond with G4421 and side-chain contacts to U4526 (Fig. 3a, f). In the N-terminal direction of the NC preceding L725, one face of the long α-helical segment of the NC makes numerous contacts with nucleotides lining the ribosome exit tunnel and PF846 (Fig. 3a). Nascent-chain residues L718, L721, and I722 contact 28S rRNA nucleotides U4525, A4419, A3887, and G3883 (Fig. 3a, g–i), and residues L714, I717, and L718 engage in multiple interactions with PF846 through van der Waals forces (Fig. 3j-l). These interactions are essential for the PF846-induced translation termination as reflected by the mutagenesis results (Fig. 3e and Supplementary Fig. 10e, g). In the distal region of the ribosome exit tunnel (i.e., away from the PTC and further toward the NC N terminus), the NC makes contacts with ribosomal protein uL4 (residues W67 and R71) and uL22 (residues H133 and I136), along with nucleotide C2781 in 28S rRNA (Fig. 3a and Supplementary Fig. 10a–d). However, mutational analyses revealed that ribosome contacts to NC residues L705–L711 are less important for the inhibition of translation termination (Supplementary Fig. 10f).

To further test the importance of contacts of the NC to PF846 and the distal region of the ribosome exit tunnel, we constructed two cell lines expressing nanoluciferase–CDH1–NPN* reporters harboring two representative NC mutations, I717A and Q706A. Whereas the I717A mutation decreased the overall magnitude of PF846-induced stalling and increased the IC_50_ ~2.5× compared to the CDH1–NPN* sequence, the Q706A mutation had no effect on overall inhibition (Supplementary Fig. 10h). Taken together, the structural and mutagenesis results reveal that PF846-induced inhibition of translation termination requires a NC sequence distributed over 15 amino acids (residues L714–N728).

### eRF1 conformation in the PF846-stalled termination complex

**Fig. 4:**
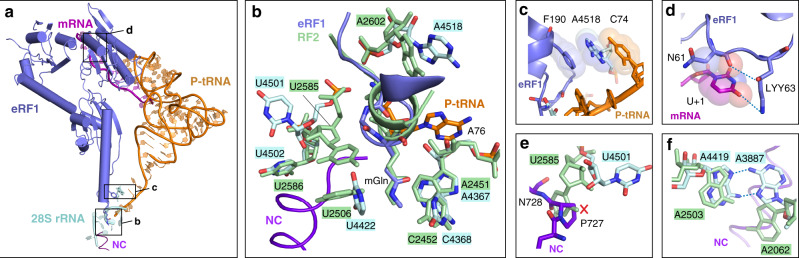
PTC rearrangements in the PF846-stalled termination complex. **a** Overview of the mRNA decoding center and PTC with eRF1 (slate blue), P-site tRNA (orange), mRNA (magenta), 28S rRNA (light cyan), nascent chain (purple), and PF846 (cyan). Essential eRF1 motifs are highlighted in boxes with letters corresponding to the panel labels. **b** Comparison of the PTC from a bacterial RF2 ribosome complex (PDB code 6c5l^11^, pale green) and in the PF846-stalled termination complex. **c** eRF1-mediated stacking interactions within the PTC, showing eRF1 residue F190, 28S rRNA nucleotide A4518, and C74 of P-site tRNA. Spheres represent van der Waals radii of C, N, and O atoms. **d** Interaction network of U + 1 in the UAA stop codon (magenta) with the eRF1 NIKS motif (slate blue). Interactions proposed to stabilize the stop codon are indicated with dashed lines for hydrogen bonds and spheres for van der Waals radii. 4*R*-hydroxylysine (LYY63) at residue 63 in eRF1 is shown. **e** Comparison of the position of 28S rRNA nucleotide U4501 with *Ec* 23S rRNA nucleotide U2585 in the RF2-bound bacterial ribosome. The steric clash with the PF846-stalled NC is highlighted with a red “X”. **f** Comparison of the positions of A3887 and A4419 with the respective nucleotides in the RF2-bound bacterial ribosome (*Ec* A2062 and U2503), showing dashed lines for hydrogen bonds.

Although structurally different, translation RFs from bacteria to eukaryotes all recognize the nucleotides in stop codons and catalyze peptide release^20–22^. They possess a highly conserved GGQ sequence motif that resides at the tip of a short α-helix and points directly into the PTC (Fig. 4a). The Gln residue is modified to *N*^5^-methyl-Gln (mGln) in both the bacterial and eukaryotic domains^23^. Computational analysis and recent structural studies of bacterial RF2 showed that this methylation can enhance the correct positioning of the Gln residue in the PTC catalytic site during translation termination^11,24^. However, in eukaryotes, the positioning of mGln remains unclear due to the lack of a high-resolution structure of a termination complex^9,10,21,25^. Here, in the PF846-stalled translation termination RNCs, we clearly resolved the density for the mGln of eRF1 (Supplementary Fig. 11a). In bacteria, the *N*5 methylation increases the van der Waals interactions with 23S rRNA nucleotides in the PTC, including *Ec* U2506, *Ec* A2451, and *Ec* A2452 (*Ec*, *Escherichia coli* numbering, Fig. 4b)^11,26^. In PF846-stalled translation termination complex, mGln adopts the same conformation and establishes multiple interactions with the equivalent PTC residues in human 28S rRNA (Fig. 4a–c and Supplementary Fig. 11a).

In the present structure, eRF1 docks in the mRNA decoding site, positioning a highly conserved NIKS motif (Asn–Ile–Lys–Ser) located in the N-terminal domain to recognize the stop codon (Fig. 4a, d)^27^. During translation termination, eRF1 is thought to directly interact with the uridine at position 1 in the stop codon (U1) by means of a post-translationally modified lysine (residue K63 in the NIKS motif) hydroxylated at the C4 carbon^28^. This modification increases translation termination efficiency by reducing stop codon read-through^29^. Although prior structures modeled K63 within hydrogen-bonding distance of U1^9,10^, the limited resolution of the cryo-EM density precluded modeling of the 4-hydroxylation. In the present structure, we were able to model 4*R*-hydroxylysine at residue 63 in the cryo-EM density, with the hydroxyl group hydrogen bonding to the backbone carbonyl of asparagine 61 in the NIKS motif and the side-chain ε-amine hydrogen bonding to the O4 carbonyl in U1 (Fig. 4d and Supplementary Fig. 11b).

### rRNA elements critical for PF846-induced inhibition of termination

**Fig. 5:**
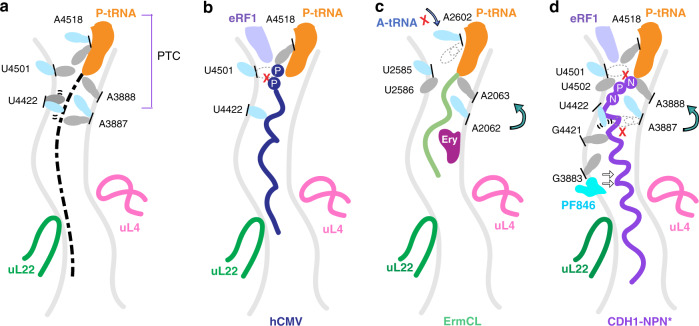
Features of PF846-mediated inhibition of translation termination. **a** Schematic of common translation stalling features, with 28S rRNA nucleotides showing conformational changes (gray, translation-competent conformation; light cyan, inactive conformation). Nucleotides are shown with human numbering, with the corresponding *E. coli* numbering in parentheses as follows: A4518 (*Ec* A2602), U4501 (*Ec* U2585), U4422 (*Ec* U2506), A3888 (*Ec* A2063), and A3887 (*Ec* A2062). **b** Schematic showing hCMV nascent-chain-induced stalling of translation termination^10^. The critical amino acid P–P motif for hCMV-mediated stalling is indicated. U4501 is flipped away from the PTC to avoid a steric clash with the C-terminal motif, leading to inhibition of PTC catalysis. **c** Schematic representing drug-dependent stalling of ErmCL in bacteria^30,33^. A2602 blocks the accommodation of A-site tRNA, and U2585 flips away, inactivating the PTC. A2062 in the ribosome exit tunnel exhibits a distinct conformation due to the geometry of the NC and transmits the stalling signal back to the PTC through the neighboring nucleotides including A2063. **d** Model for PF846-stalled translation termination. The ribosome exit tunnel nucleotides essential for stalling are labeled. Stalling is propagated from PF846 back to the PTC by contacts with and rearrangements of multiple nucleotides. In panels **b**–**d**, collisions with nucleotides in their active conformation are shown by a red “X”.

The positioning of eRF1 in both the mRNA decoding site in the small ribosomal subunit and the PTC in the large ribosomal subunit indicates that eRF1 is bound in an active conformation^9–11^. However, rearrangements in the PTC and ribosome exit tunnel could explain the slow hydrolysis of peptidyl-tRNA and trapping of the nascent chain by PF846. In bacteria, two universally conserved rRNA nucleotides, *Ec* A2602 and *Ec* U2585 (A4518 and U4501 in human 28S rRNA, respectively) are required for peptide release^30–32^. In the present structure, A4518 occupies a similar position to *Ec* A2602 in the bacterial translation termination complex^11^, and stabilizes the positioning of the GGQ catalytic loop by stacking between F190 in eRF1 and the C74 of P-site tRNA (Fig. 4c). However, nucleotide U4501 (*Ec* U2585) is rotated by 90° away from the PTC, to avoid a steric clash with P727 in the nascent chain (Fig. 4b, e and Supplementary Fig. 11a). Interestingly, the same change in the position of U4501 is observed both in human cytomegalovirus (hCMV)-arrested translation termination complexes^10^ (Fig. 5a, b and Supplementary Fig. 12a, b) and macrolide-dependent stalling of ErmCL in bacteria^33,34^ (Fig. 5c, Supplementary Fig. 12a, b). In the ribosome exit tunnel, universally conserved nucleotides A3887 and A4419 (*Ec* A2062 and *Ec* A2503, respectively) form a noncanonical A–A base pair (Fig. 4f) previously found to be essential in macrolide-dependent ribosome stalling (Fig. 5c)^30,33,35^. This base pair makes multiple interactions with NC residues critical for PF846-stalled translation termination (Fig. 3b, d, g). Reflecting the sequence specificity of stalling, A3887 adopts a conformation that avoids a steric clash with the NC, distinctly different from that observed in hCMV-induced stalling (Supplementary Fig. 12c). Finally, U4422 (*Ec* U2506), which is known to move positions as part of the induced fit of the PTC required for peptide bond formation^36,37^, is highly mobile based on the cryo-EM density, forming different extents of contacts to the nascent chain and mGln185 of eRF1 (Supplementary Fig. 12d–h). This suggests that U4422 may be less essential in PF846-mediated trapping of the CDH1–NPN NC on the ribosome.

## Discussion

We recently reported that drug-like compounds can selectively stall translation during nascent peptide elongation, by impeding the movement of the ribosomes along the mRNA in a nascent-chain-dependent manner^4,7^. These compounds exert their selective effect while bound in the ribosome exit tunnel, despite the fact that all protein nascent chains transit this space in the ribosome. Unexpectedly, these same small molecules can also block eukaryotic translation termination on specific nascent-chain sequences^4,7^. Here, we show that these molecules employ a unique mechanism of action to block translation termination and trap the protein nascent chain on the ribosome.

Using cryo-EM, we identified two different states of PF846-stalled RNCs, the first in a rotated state similar to that observed for PF846-stalled translation elongation (Figs. 1b and 2a)^7^. The second in the nonrotated state represents a stalled translation termination complex (Fig. 1c and Fig. 2b). These structures suggest a two-step model for PF846-mediated inhibition of translation termination. First, PF846 induces a slowdown in translation before the stop codon enters the mRNA decoding site (A site), similar to what is observed for PF846-mediated stalling of translation elongation^7^ (Fig. 1b and Supplementary Fig. 3a–c). Subsequently, after the stop codon is recognized by eRF1, PF846 inhibits hydrolysis of the NC from the P-site tRNA in a NC sequence-dependent manner (Fig. 2c, Fig. 3e, and Supplementary Figs. 7, 8 and 10). Unexpectedly, some released NCs, including all of the CDH1–NPN* variants and CDH1–GCV*, remain trapped on the ribosomes in vitro even in the absence of PF846 (Fig. 2c, Fig. 3e, Supplementary Fig. 8b, and Supplementary Fig. 10e). Thus, the presence of released nascent chains in RNC complexes is not due to trapping by PF846, but is an inherent biochemical property of certain NC sequences. Notably, PF846 only weakly inhibits translation of the CDH1–GCV* sequence in cells (Fig. 1a). Thus, the NC we see released from P-site tRNA but trapped in the ribosome exit tunnel in vitro is not likely to represent the situation in the cellular environment, which includes pulling forces exerted by chaperones or cotranslational protein folding^19,38^.

Stalling of translation termination by PF846 involves mechanisms distinct from those seen in bacterial and other eukaryotic stalling systems (Fig. 4 and Fig. 5)^10,39–42^. For example, the recently described bacterial termination-specific inhibitor apidaecin (Api), a 19-amino-acid long proline-rich antimicrobial peptide, binds to the ribosome exit tunnel when no NC is present and traps the release factors (RF1 or RF2) in the A site by stable interactions with its C-terminal amino acid^43–45^. By contrast, PF846-stalled termination suppresses translation termination prior to eRF1-directed hydrolysis of the NC–tRNA ester bond. Like the PF846-arrested NC, the hCMV stalling peptide forms an α-helix in the exit tunnel and silences PTC activity, thus affecting the release activity of an otherwise productively positioned GGQ of eRF1 (Fig. 5b)^9,10,46^. However, hCMV-mediated translation termination is mainly inhibited by two C-terminal prolines (Fig. 5b)^9,10,46^. By contrast, the C-terminal NPN motif by itself is insufficient for PF846-induced stalling (Fig. 3). Instead, PF846 traps the NC in the ribosome exit tunnel by making contacts along one face of a longer NC α-helix (Fig. 5d and Supplementary Fig. 13). Mutation of these residues leads to diminished or abolished stalling (Fig. 3e and Supplementary Fig. 10e). Additional contacts between the NC and nucleotide A3887 (*Ec* A2062) in the exit tunnel are required for PF846-induced stalling (Fig. 3), similar to macrolide-dependent stalling in bacteria (Fig. 5c, d)^30^. However, in contrast with macrolide-dependent stalling in bacteria, PF846 does not lead to nucleotide rearrangements that would block tRNA and RF access to the A site in the PTC^33,47^ (Fig. 5c, d). In the human genome, approximately 447 proteins encode NPN-like motifs next to the stop codon^7^, including one example of NPN* (Methods). However, the distributed sequence and structural determinants important for PF846-induced stalling of translation termination (8 of the C-terminal NC amino acids distributed over 15 residues within the ribosome exit tunnel) indicate that molecules like PF846 could be used for selective inhibition of translation for new therapeutic targets. Taken together, our experimental data elucidate the mechanism of drug-like compound-stalled translation termination in human ribosomes, providing insights into the sequence specificity of compounds like PF846 that can aid development of these compounds for therapeutic purposes.

## Methods

### DNA constructs and in vitro transcription

The DNA plasmid encoding the mRNA for the CDH1–NPN* stalling construct was previously described^7^, which includes CDH1 residues 586–725, followed by amino acids NPN and a UAA stop codon (Supplementary Fig. 2a). Point mutations in the CDH1–NPN* protein nascent-chain sequences were generated through “around-the-horn” cloning^48^ using primers with overhangs (Supplementary Table 1). Plasmids used to construct lentiviral vectors encoded nanoluciferase with an N-terminal 3×FLAG peptide, and the CDH1 stalling sequence with different termination motifs or mutations (GCV*, NPN*, Q706A, and I717A), along with the CDH1 3’-untranslated region (3’-UTR) (Supplementary Fig. 1a). The DNA fragment was assembled using overlap extension polymerase chain reaction (PCR) with primers shown in Supplementary Table 1. The resulting PCR product was subsequently inserted using Gibson Assembly Master Mix (NEB, E2611L) into the lentiviral vector CD813A (System Biosciences) encoding the 5′ untranslated region of human β-globin (*HBB* 5′-UTR).

DNA templates for in vitro transcription were amplified by PCR using primers encoding a T7 RNA polymerase promoter and a poly-A tail (Supplementary Table 1). All PCR products were purified via QIAquick Gel Extraction Kit (Qiagen, 28115) before their use in in vitro transcription reactions. Messenger RNAs were transcribed using T7 RNA polymerase prepared in-house. Reactions were set up with 20 mM Tris-HCl pH 7.5, 35 mM MgCl_2_, 2 mM spermidine, 10 mM DTT, 1 U/mL inorganic pyrophosphatase (ThermoFisher, EF0221), 7.5 mM each NTP, 0.2 U/L SUPERaseIn RNase Inhibitor (ThermoFisher, AM2696), 0.1 mg/mL T7 RNA polymerase, and 40 ng/μL DNA. After 4 h of incubation at 37 °C, 0.1 U/μL RQ1 RNase-free DNase (Promega, M6101) was added to the reactions, followed by another incubation at 37 °C for 30 min to remove the template DNA. RNA was precipitated overnight at −20 °C after adding 1/2 volume of 7.5 M LiCl/50 mM EDTA, and the resulting pellet was washed with cold 70% ethanol and dissolved with RNase-free water. RNAs were purified using Zymo RNA Clean and Concentrator kit following the manufacturer’s instructions (Zymo research, R1017) before use in in vitro translation reactions.

### Generation of stable cell lines

Lentiviruses encoding the various CDH1-derived stalling sequences were generated using HEK293T cells in 10-cm dishes. Cells grown to a confluence of 80% were transfected with the CD831A plasmids encoding the stalling sequences described above, together with helper plasmids PsPAX2 and pCMV-VSV-G (Addgene), using the TransIT-LT1 transfection reagent (Mirus Bio, MIR 6000) following the manufacturer’s instructions. Lentiviruses were harvested and filtered with 0.22-μm filters after 48 and 72 h. To generate stable cell lines, 1 mL of each virus was added to HEK293T cells seeded in a 6-well plate at a density of around 80–90% confluence, along with 10 μg/μL of polybrene (Millipore, TR-1003-G). After 24 h, the cells were treated with 4 μg/mL puromycin (Gibco, A1113803) for 4 days and split in DMEM media (Dulbecco’s Modified Eagle Medium, Invitrogen) with 10% FBS (Tissue Culture Biologicals) before use. Primers and gene blocks used for cell line generation are given in Supplementary Table 2.

### Luciferase reporter assay

To determine the steady-state conditions for PF846-induced stalling of nanoluciferase reporters, the stable cell lines expressing nanoluciferase reporter genes fused to the various CDH1 stalling sequences were treated with 5 μM PF846 in 0.1% DMSO or 0.1% DMSO as the control. Luciferase activity was assayed after 1.5, 7, 10, 20, 27, and 43 hr using the Nano-Glo^®^ Luciferase Assay System (Promega, N1120). To determine the concentration dependence of PF846 inhibition of nanoluciferase reporters, the cell lines were treated with 0.1% DMSO and different concentrations of PF846 in 0.1% DMSO (1, 3, 5, 7, 10, 20, 50, and 100 μM) for 20 h. After treatment, nanoluciferase activity was assayed as described above. The IC_50_ calculated here is likely an upper bound due to the accumulation of secreted nanoluciferase in the cell culture media^49^.

### In vitro translation reactions

Extracts from HeLa cells were made as described previously^4,7^. For the in vitro translation reactions to assess mutations in the nascent-chain sequence, a 30-μL final volume was used for each mRNA, which contained 15 μL cell lysate and buffer with a final concentration of 20 mM HEPES pH 7.4, 120 mM KOAc, 2.5 mM Mg(OAc)_2_, 1 mM ATP/GTP, 2 mM creatine phosphate (Roche), 10 ng μL^−1^ creatine kinase (Roche), 0.21 mM spermidine, 0.6 mM putrescine, 2 mM TCEP (tris(2-carboxyethyl)phosphine), 10 μM amino acid mixture (Promega, L4461), 1 U μL^−1^ murine RNase inhibitor (NEB, M0314L), 600 ng of mRNA, and 50 μM PF846 in 1% DMSO as control. Translation reactions were incubated for 23 min at 30 °C, followed by centrifugation for 5 min at 20,000*g* to remove the cell debris. The supernatant was applied to a 50% sucrose cushion (260 μL) prepared with cushion buffer (25 mM HEPES-KOH pH 7.5, 120 mM KOAc and 2.5 mM Mg(OAc)_2_, 1 M sucrose, 1 mM DTT, and 50 μM PF846) and centrifuged for 1 hour at 603,000*g* using a MLA-130 rotor (Beckman Coulter) at 4 °C. The pellet was suspended in ice-cold RNC buffer (20 mM HEPES pH 7.4, 300 mM potassium acetate, 5 mM magnesium acetate, 1 mM DTT, and 0.2 mM PF846). The RNC samples were then used for western blot analysis.

For the time-course assay in Fig. 2c and Supplementary Fig. 8, the RNCs were assembled with different nascent chains from in vitro translation reactions in the presence (+) or absence (–) of 50 μM PF846 for 23 min at 30 °C. We then aliquoted these reactions into different tubes and incubated at room temperature for different times as indicated in Fig. 2c and Supplementary Fig. 8 legend. Puromycin treatment was conducted by adding a final concentration of 1 mM puromycin into the IVT reaction followed by incubation at room temperature for 1 h. Sucrose cushions were used to isolate ribosomes, followed by western blotting as described above. Uncropped gel images for Fig. 2c and Supplementary Fig. 8 are available as Source Data.

### RNase A treatment and western blot

To assess the total amount of stalled nascent chains, RNC samples purified from sucrose cushions, as described above, were treated with 100 μg mL^−1^ DNase-free RNase A (ThermoFisher, EN0531) at 37 °C for 50 min followed by western blot with monoclonal Anti-FLAG M2-Peroxidase (HRP) antibody (Sigma, A8592) for the CDH1-derived nascent chains (1:10,000 dilution), as well as with an anti-RPLP0 antibody (Bethyl Laboratories, A302-882A) used as a loading control (1:8000 dilution). In order to visualize tRNA-bound stalled nascent chains, the RNC samples from the above sucrose cushions were heated at 55 °C for 5 min in the presence of 1× Laemmli Sample buffer (Bio-Rad, 1610737). Subsequently, these samples were resolved on NuPAGE 4–12% Bis–Tris protein gels (ThermoFisher Scientific, NP0323PK2) prior to western blot analysis^50^.

### Purification of stalled RNCs and cryo-EM grid preparation

An in vitro translation reaction of 1.5 mL programmed with mRNA encoding the CDH1–NPN* sequence was incubated with 50 μM PF846 at 30 °C for 23 min and then centrifuged at 20,000*g* for 5 min. The supernatant was incubated with 50 μL of anti-FLAG M2 agarose beads (Sigma, A2220) for 1 h at room temperature with gentle mixing. All the following purification steps were conducted at room temperature to avoid nonspecific binding of 80S particles to the beads, unless specifically noted. The 3×FLAG-tagged RNCs bound to the anti-FLAG beads were washed 3 times with 200 μL of RNC buffer, then 3 times with 200 μL of RNC wash buffer plus 0.1% Triton X-100, followed by 3 times with 200 μL of RNC buffer plus 0.5% Triton X-100, and finally washed twice with 200 μL of RNC buffer. A final concentration of 0.2 mg mL^−1^ 3×FLAG peptide (Sigma, F4799) in the RNC buffer was used to release the RNCs from the beads. The resulting RNCs were pelleted through a sucrose cushion as described above, and resuspended in ice-cold RNC buffer that was immediately used for making cryo-EM grids.

The concentration of the purified RNC used for cryo-EM grid preparation was 50 nM. Approximately, 3.2 μL of RNCs were incubated on plasma-cleaned 300-mesh holey carbon grids (C-flat R2/2, Electron Microscopy Science) for 1 min, on which a homemade continuous carbon film was precoated. Grids were blotted for 3 s under 10% humidity at 4 °C and plunge-frozen in liquid ethane using a FEI Vitrobot.

### Cryo-EM data collection and processing

Cryo-EM data were collected using a Titan Krios electron microscope (FEI) equipped with a K2 Summit direct detector and GIF Quantum filter (Gatan) at 300 kV and running SerialEM software (Table 1)^51^. The total exposure time was 9 s, with a total dose of 50 electrons Å^−2^ (frame dose 1.3 electrons Å^−2^). The frames in the resulting movies were corrected for motions using MotionCor2 with FtBin 2 and dose weighting^52^. The subsequent processing was performed in RELION 3.1^53^. For the initial steps of image processing, the data were binned by a factor of 4. After 2D classifications to remove images with ice or other contaminants, 3D classification was used to remove nonribosomal particles (82,787 particles). After 3D refinement, an additional 3D classification was performed to separate the ribosomes in the rotated state (26,463 particles) and nonrotated state (57,324 particles) (Supplementary Fig. 2b). After one round of CTF refinement and Bayesian polishing^54^, particles of each class were further processed by focused refinements with individual masks applied (Supplementary Fig. 2b)^55^. Binary masks with smoothed edges were generated with the “relion_mask_create” tool in RELION. Postprocessing and B-factor sharpening implemented in RELION 3.1 was applied to the final maps. EMBFACTOR was also used to apply certain B factors in order to better visualize the density of certain parts of the RNC maps^56^. The reported resolutions for all maps are based on the FSC cutoff criterion of 0.143^57,58^. Local resolution estimation was performed using Resmap^59^.

**Table 1:** Cryo-EM data collection, refinement, and validation statistics of PF846-stalled termination complexes.

	Classical RNC (EMD-22085, PDB-6XA1)	Rotated RNC (EMD-22086)
*Data collection and processing*		
Magnification	43,478	43,478
Voltage (kV)	300	300
Electron exposure (e^–^/Å^2^)	50	50
Defocus range (μm)	−1.0 to −2.0	−1.0 to −2.0
Pixel size (Å)	1.15	1.15
Symmetry imposed	C1	C1
Initial particle (no.)	183,759	183,759
Final particle (no.)	57,324	26,463
Map resolution (Å)	2.7	2.9
FSC threshold	0.143	0.143
*Refinement*		
Average resolution (Å)	2.7 Å (60 S)/2.8 Å (40 S)/2.8 Å (40S_eRF1_tRNA_mRNA)	2.9 Å (60 S)/3.1 Å (40 S)/3.1 Å (40 S_tRNA_mRNA)
Map-sharpening *B* factor (Å^2^)	−43	−48
Initial model used (PDB code)		6OLE
40S subunit	6qzp	
60S subunit	6qzp	
eRF1_tRNA_mRNA	6d90	
*R.m.s. deviations*		
Bond lengths (Å)	0.014	0.013
Bond angles (°)	1.103	1.267
*Validation*		
Clashscore	3.36	4.70
Poor rotamers (%)	0.53%	0.94%
*Ramachandran statistics*		
Favored (%)	93.85%	92.46%
Allowed (%)	6.03%	7.38%
Outliers (%)	0.53%	0.17%

### Model building and refinement

The atomic models of the human 80S ribosome (PDB: 6qzp)^14^ were used as the starting point and manually adjusted and refined in COOT^60^ using the experimental cryo-EM maps. For the nonrotated RNC, eRF1, and P-tRNA was initially modeled using the mammalian 80S ribosome with eRF1 and P-site tRNA bound (PDB: 6D90)^61^. The CDH1–NPN nascent chain was modeled manually into the density using COOT^60^ (CDH1–NPN nascent-chain sequence: EAGLQIPAILGILGGILALLILILNPN). The post-translational modification of K63 in eRF1, 4-hydroxylysine (LYY63), was generated using ChemDraw 19.0 (PerkinElmer) to create the SMILES string, which was then used as the input to phenix.elbow^62^. Subsequent adjustments to the eRF1 model in the vicinity of the 4-hydroxylysine were made using phenix.real_space_refine^62–64^ and the 40S subunit focused–refined map. The post-translational modification of Q185 to mGln185 used the standard .cif library in CCP4^65^.

To model the rotated-state RNC structure, we used the previously reported PF846-stalled RNC model (PDB: 6OLE)^7^ docked into the cryo-EM map by rigid-body fitting followed by refinement in Phenix^63,64^. Both RNC structures were refined using Phenix (phenix.real_space_refine) with RNA secondary structure restraints imposed^63,64^. Model refinement and validation statistics are provided in Table 1.

### Secondary structure modeling

Modeling of the secondary structure of the PF846-sensitive PCSK9 stalling sequence was carried out using Phyre2^66^.

### NPN-patterned motif searching in human genome

To investigate the prevalence of NPN-like motifs adjacent to the stop codon in the human genome, we first downloaded the annotated protein-coding sequences (CCDS_protein.current.faa) from the NCBI website http://ftp.ncbi.nih.gov/pub/CCDS/current_human. We then use the following commands to identify the number of human proteins with the motif as described in^7^.

>grep -B 2 CCDS grep -B 2 CCDS_protein.current.faa > temp1

>awk ‘/>CCD/{if (NR! = 1)print “”}{printf $0}END{print “”;}’ temp1 | grep -o ‘…$‘ > temp2

> grep ‘[DNHY][PV][A-Y]’ temp2 | wc -l

### Figure preparation

Figures were prepared using UCSF Chimera^67^, UCSF ChimeraX^68^, and PyMOL (Schrödinger).

### Reporting summary

Further information on research design is available in the Nature Research Reporting Summary linked to this article.

## Supplementary information

Supplementary Information

Peer Review

Reporting Summary

## Data Availability

The data that support the findings of this study are available from the corresponding author upon reasonable request. The cryo-EM maps have been deposited with the Electron Microscopy Data Bank under the accession codes EMD-22085 (nonrotated RNC with eRF1) and EMD-22086 (rotated RNC). Atomic coordinates have been deposited in the Protein Data Bank with accession codes 6XA1 (nonrotated RNC with eRF1). Source data are provided with this paper.

## Source data

Source Data
