# Supplementary material for: Selective inhibition of human translation termination by a drug-like compound
Source: Nat Commun. 2020 Oct 2;11:4941. doi: 10.1038/s41467-020-18765-2 (PMC7532171; doi:10.1038/s41467-020-18765-2)
Supplement: Supplementary file 1 — Supplementary Information [file 41467_2020_18765_MOESM1_ESM.pdf]

Title: Selective inhibition of human translation termination by a drug-like compound

Authors: Wenfei Li[1,2,3], Stacey Tsai-Lan Chang[1,2], Fred. R Ward[1] and Jamie H. D. Cate[1,2,3,4]*

[1]Department of Molecular & Cell Biology, University of California, Berkeley, California 94720, USA. [2]Innovative Genomics Institute, University of California, Berkeley, California 94720. [3]Molecular Biophysics and Bioimaging Division, Lawrence Berkeley National Laboratory, Berkeley, California 94720, USA. [4]Department of Chemistry, University of California, Berkeley, California, USA. *e-mail: j-h-doudna-cate@berkeley.edu

Supplementary Information

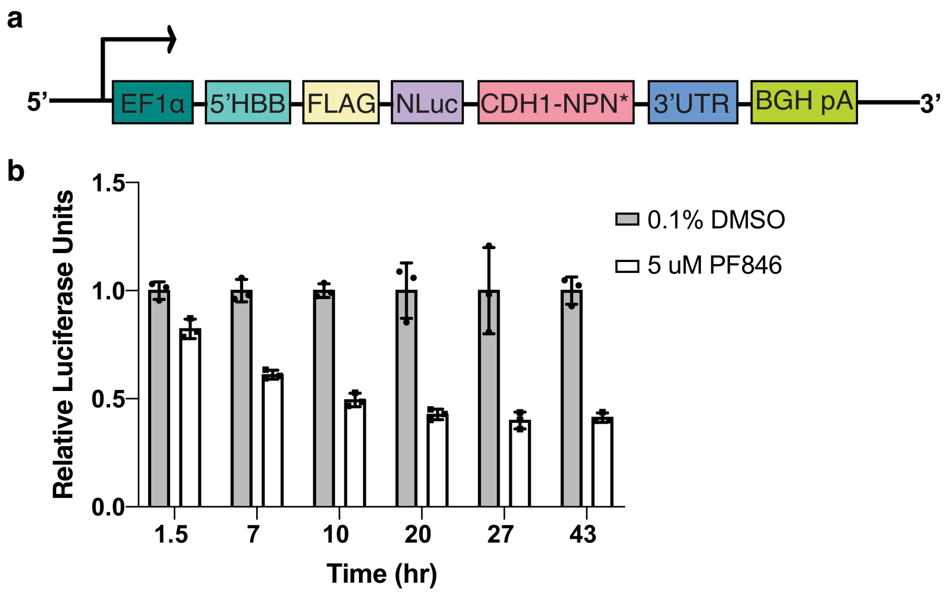

Supplementary Fig. 1 | Inhibition of translation termination by PF846 in cells. (**a**) Schematic representation of the CDH1-NPN* lentiviral construct used for making stable cell lines. (**b**) Luciferase reporter assays using the CDH1-NPN* cell line at different time points after treatments with 0.1% DMSO (grey bars) or 5 μM PF846 (white bars). Bars show mean ± s.d., n = 3 independent experiments. Source data for (**b**) is available in **Supplementary Data Set 1**.

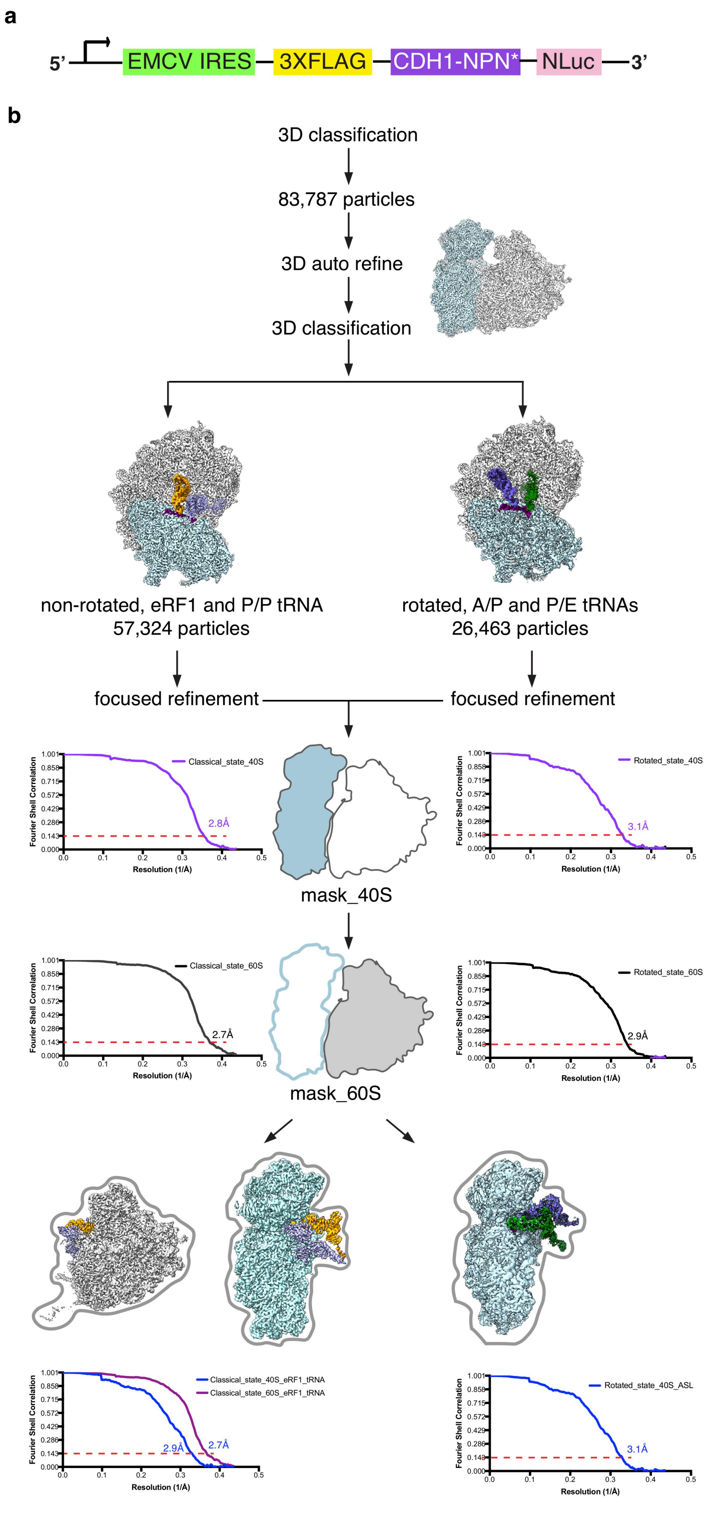

Supplementary Fig. 2 | Cryo-EM data processing of PF846-stalled termination complexes. (**a**) Schematic representation of the DNA construct used to prepare PF846-

stalled termination complexes. (**b**) Cryo-EM data processing workflow. Particle numbers at different steps are indicated. Two main populations were obtained after 3D classification. For the non-rotated state, focused refinements involved masking of the 40S subunit or 60S subunit individually, or the 40S subunit with eRF1, mRNA and tRNA, or the 60S subunit with eRF1 and tRNA. For the rotated state, focused refinements involved masking of the 40S subunit or 60S subunit individually, or the 40S subunit with mRNA and tRNA ASLs (anticodon stem loops). Final FSC curves of stalled RNCs in the non-rotated state and rotated state are presented, with the “gold standard” value of 0.143 used to define the resolution.

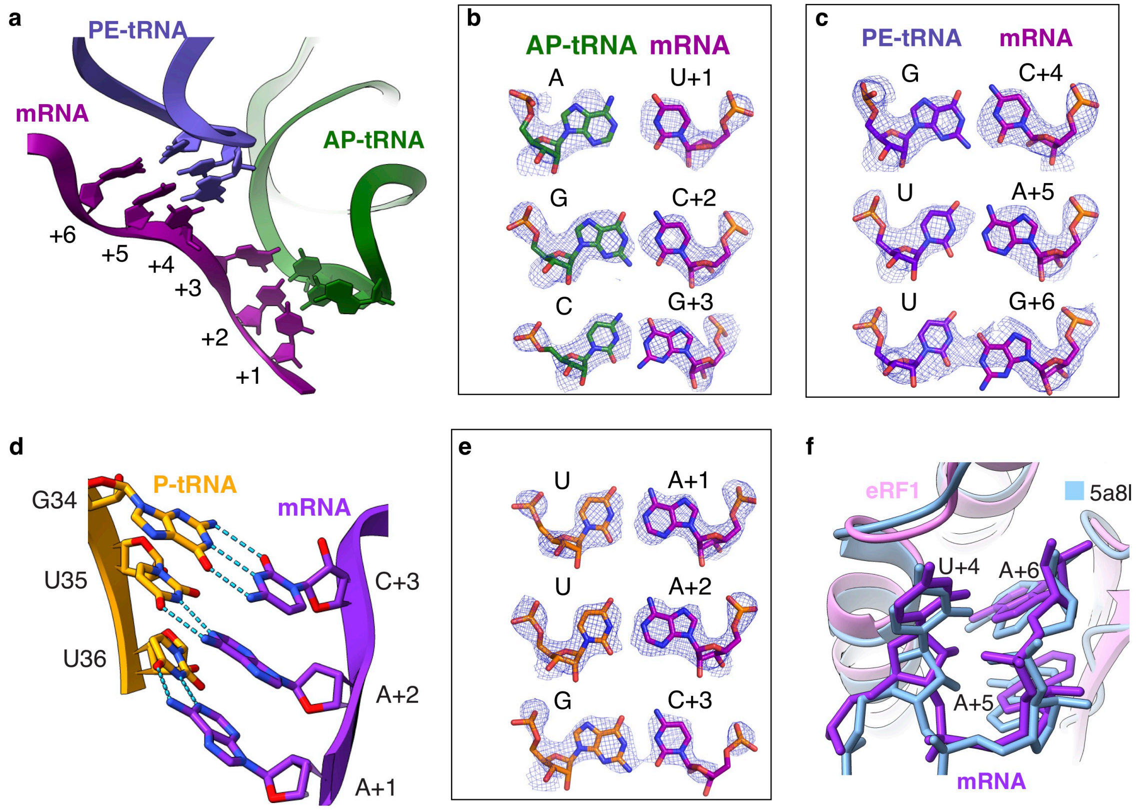

Supplementary Fig. 3 | Analysis of the mRNA codon-tRNA anticodon stem loop (ASL) interactions. (**a**) Model of the ASLs from the rotated RNC, with mRNA colored in magenta, P/E-site tRNA in dark purple and A/P-site tRNA in green. (**b-c**) Models of the base pairs between A/P-site tRNA anticodon nucleotides and mRNA codon nucleotides. Note that the individual nucleotides cannot be modeled with either pyrimidine or purine bases. The base pairs were refined into the cryo-EM density of the rotated RNC using Phenix. (**d**) Model of the P-site tRNA ASL (orange) from the non-rotated state RNC, with mRNA in magenta. The dashed lines indicate base pairing interactions. (**e**) Cryo-EM density of the P-site tRNA and mRNA in the non-rotated state, colored as in (**d**). (**f**) Superposition of the UAA stop codon with a previously-reported human termination structure (light blue) [11].

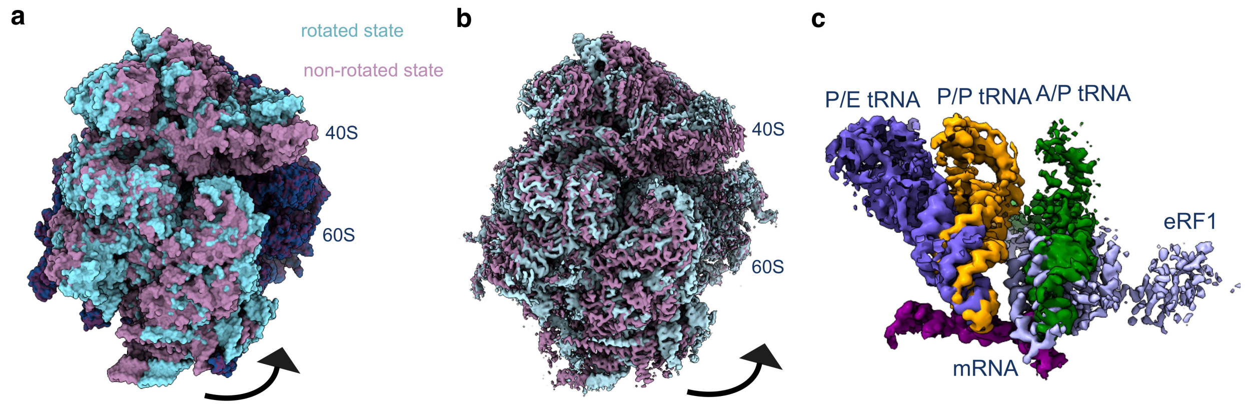

Supplementary Fig. 4 | Comparison of the two different states of PF846-stalled termination RNCs. (**a**) Comparison of the atomic model (shown with surface in ChimeraX [69]) of the 40S subunit in the rotated state (light cyan) with the non-rotated state (dark purple). Alignments were done using the model or map with 60S subunit as the reference. (**b**) Comparison of the cryo-EM density map of the 40S subunit in the rotated state (light cyan) with the non-rotated state (dark purple). (**c**) Comparisons of the ribosomal A, P, and E sites in both structural models, with A/P-site tRNA (dark green) and P/E-site tRNA (purple) from the rotated state; eRF1 (slate blue) and P/P-site tRNA (orange) from the non-rotated state.

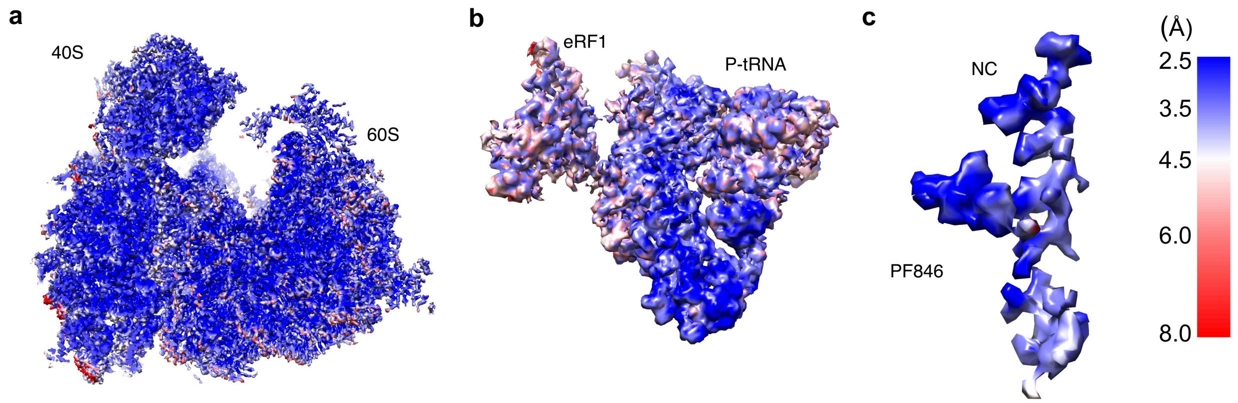

Supplementary Fig. 5 | Local resolution of cryo-EM maps of PF846-stalled termination RNCs. (**a-c**) Local resolution estimation of the non-rotated RNC with (**a**) 40S subunit and 60S subunit, (**b**) 40S subunit plus tRNA plus eRF1 and (**c**) NC plus PF846. Resolution scale in angstroms (Å) is shown to the right.

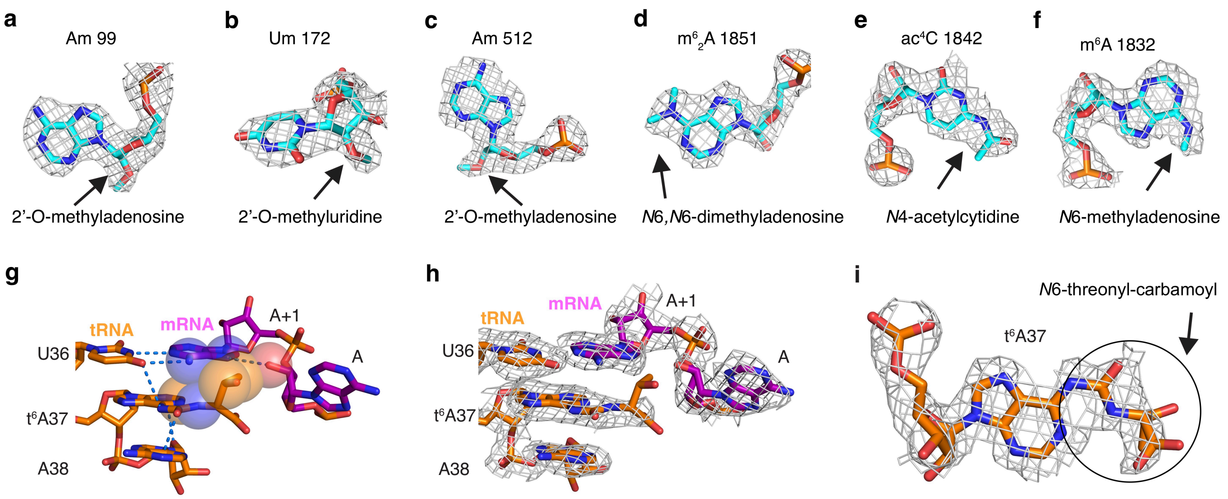

Supplementary Fig. 6 | Representative rRNA and tRNA modifications from the non-rotated state. (**a-c**) Representative 18S rRNA modifications found in the non-rotated RNC, observed in mass spectrometry experiments [16] but not in previous cryo-EM structures. (**d-f**) Representative 18S rRNA modifications observed in this study. (**g**) Interactions between mRNA codon nucleotides A and A+1 (magenta) with P-site tRNA nucleotides 36-38 (orange). Hydrogen bonds are indicated with dashed lines and van der Waals radii are shown with spheres. (**h**) Density for model shown in (**g**). (**i**) Model and cryo-EM density for *N6*-threonyl-carbamoyl adenosine at nucleotide 37 (t^6A37), a universal tRNA modification of almost all tRNAs decoding ANN codons (N = A,U,C or G) [17]. The *N*-threonylcarbamoyl group is highlighted with a circle.

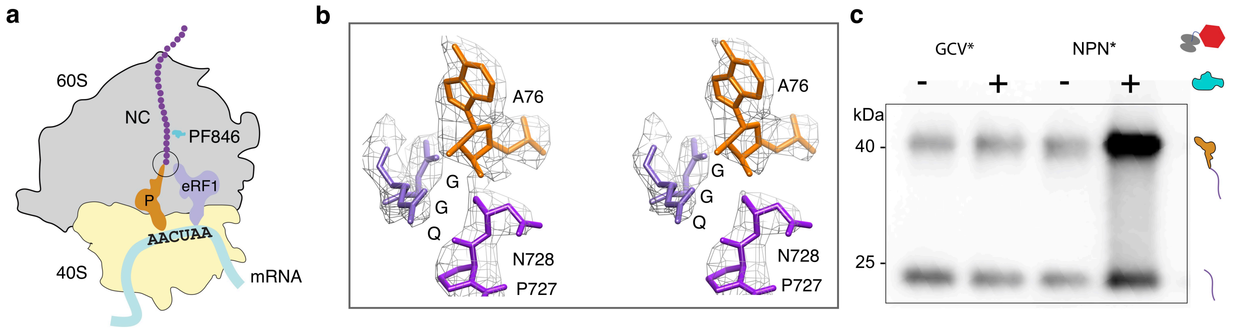

Supplementary Fig. 7 | Analysis of NC-tRNA in PF846 stalled CDH1-NPN* complex. (**a**) RNC model for PF846-stalled ribosome nascent chain complex, with ester bond region indicated with a circle. (**b**) Zoom in view of the ester bond region with low (left) and high (right) contour levels. The model for the GGQ motif of eRF1, A76 of P-site tRNA and the C terminus of the nascent chain is shown, with mesh indicating the cryo-EM density. A single threshold is used in visualizing the density map for different ligands in each panel. (**c**) Affinity purification of CDH1-GCV* and CDH1-NPN* in the absence (–) or presence (+) of PF846. Pelleted RNCs were loaded on the gel with the same volume. Experiment shown in (**c**) was repeated 3 times independently with similar results. Uncropped gel image for (**c**) is available in **Supplementary Data Set 3.**

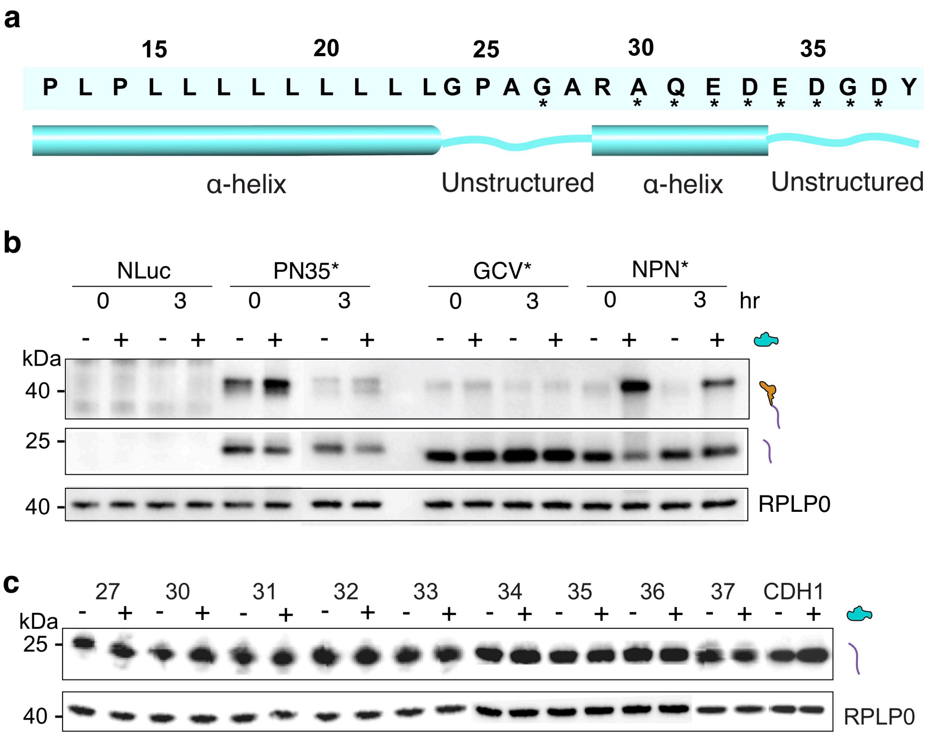

Supplementary Fig. 8 | Analysis of PCSK-derived nascent chains in PF846-stalled termination complexes *in vitro*. (**a**) Secondary structure prediction of the PCSK9 nascent chain sequence required for PF846-dependent stalling of elongation, using Phyre2 [67], with α-helices represented by cylinders and unstructured regions as curved lines. The stalling sequence of PCSK9 is shown. A break is observed from G24 to A28 which would prevent the PCSK9 sequence from forming a complete α-helix as observed for the CDH1-NPN sequence. Asterisks indicate the beginning amino acid for NPN* replacements used in translation assays. (**b**) Time course analysis of pelleted RNCs assembled with different nascent chains from *in vitro* translation reactions in the presence (+) or absence (–) of 50 μM PF846. The positions of tRNA-bound and free nascent chains are shown, with RPLP0 serving as a loading control. (**c**) Western blot

showing the effects of NPN* insertions at different positions in the PCSK9 nascent chain. *In vitro* translation reactions with DMSO (–) or with 50 μM PF846 (+) are shown. Samples were treated with RNase A, and the stalled PCSK9 nascent chains were detected by blotting with an anti-FLAG antibody, with RPLP0 serving as a loading control. Experiments shown in (**b-c**) were repeated 3 times independently with similar results. Uncropped gel images for (**b-c**) are available in **Supplementary Data Set 3** and (**d**) is available in **Supplementary Data Set 4**.

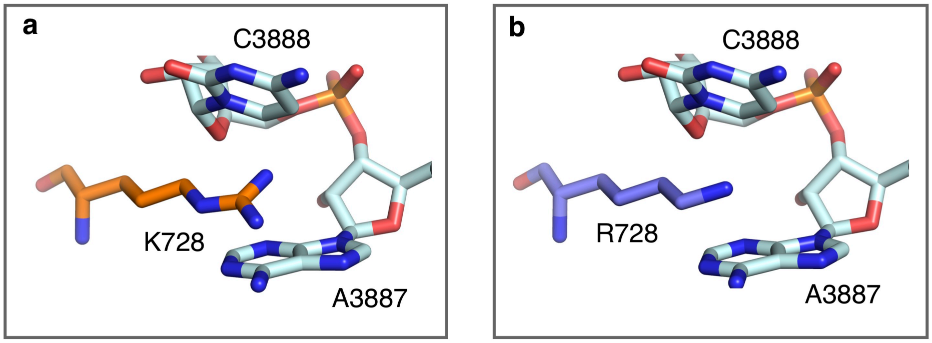

Supplementary Fig. 9 | Models of different amino acids at the CDH1-NPN C-terminal position 728. (**a-b**) The pocket formed by 28S rRNA nucleotides A3887 and C3888 can accommodate different sizes of amino acids, with Lys (K728 in **a**) and Arg (R728 in **b**) as examples.

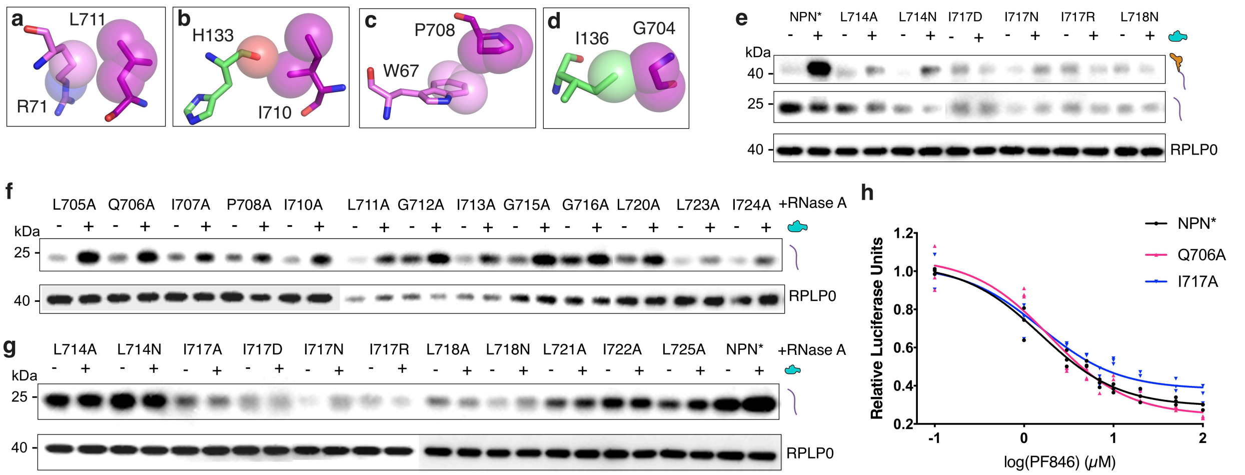

Supplementary Fig. 10 | Effects of mutations in the CDH1-NPN nascent chain on PF846-dependent inhibition of termination. (**a-d**) Interactions of ribosomal protein uL22 (light green) and uL4 (hot pink) with the nascent chain, with van der Waals surfaces shown. (**e**) Western blots of FLAG-tagged CDH1-NPN nascent chains containing single mutations, from *in vitro* translation reactions in the presence (+) or absence (–) of 50 μM PF846. The positions of tRNA-bound and free nascent chains are shown, with RPLP0 serving as a loading control. RNCs were assembled as in **Figure 3e**. (**f-g**) Western blots of CDH1-NPN nascent chains containing single mutations after treatment with RNase A. (**h**) IC_{50} values for PF846-dependent inhibition of translation termination, using stable cell lines expressing the CDH1-NPN* nascent chain (black dots) or NCs with mutations Q706A (red dots) and I717A (blue dots). Data in (**h**) show mean ± s.d., *n* = 3 independent experiments. Experiment shown in (**e-g**) was repeated 3 times independently with similar results. Source data for (**h**) are available in **Supplementary Data Set 1**. Uncropped gel images for (**e**) are available in **Supplementary Data Set 2** and (**f-g**) are available in **Supplementary Data Set 5**.

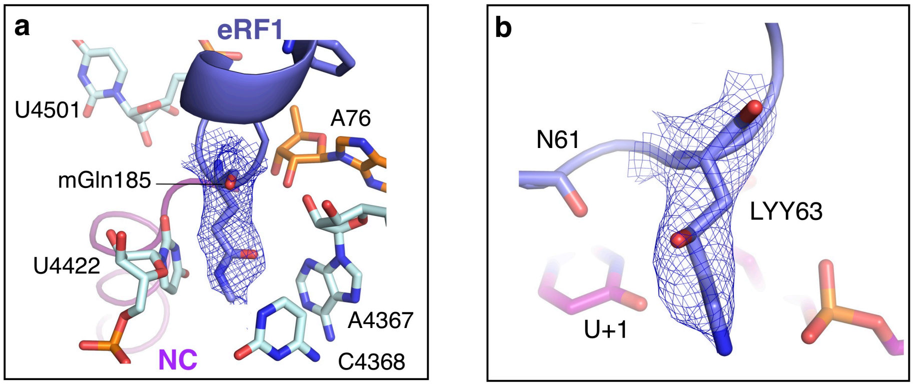

Supplementary Fig. 11 | Posttranslational modifications of eRF1 observed in the cryo-EM density map. (**a**) Close-up view of the GGQ motif within the PTC. Cryo-EM density for the methylated Gln (mGln185, slate blue) of the GGQ motif, positioned next to A76 of P-site tRNA and surrounded by PTC rRNAs, is represented with mesh. The map was sharpened with a B-factor of -40 Å^2. (**b**) The cryo-EM density for the C4 hydroxylysine 63 (LYY63) is shown in mesh. The map was sharpened with a B-factor of -40 Å^2.

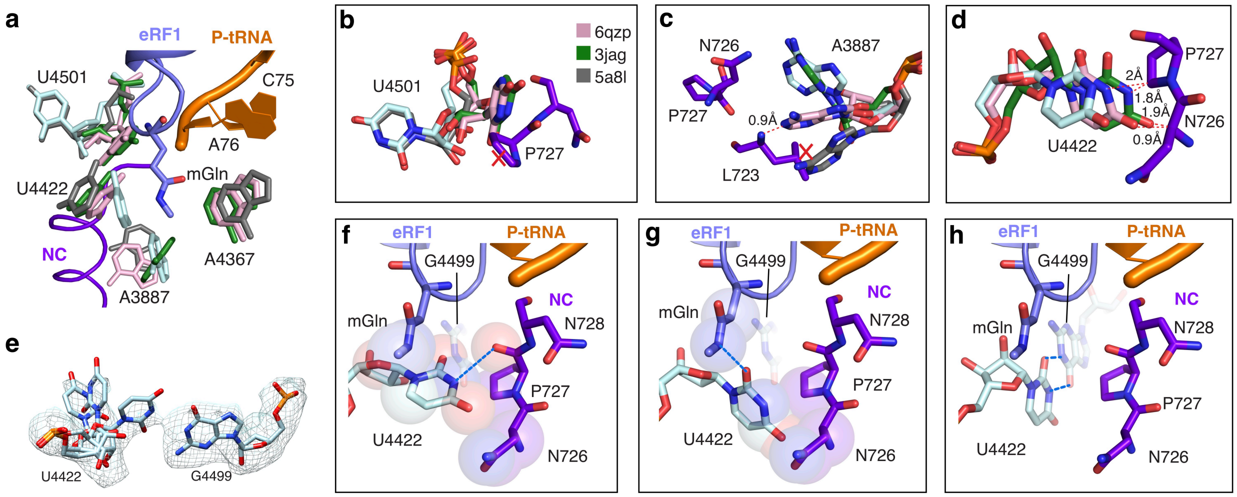

Supplementary Fig. 12 | Conformation of the PTC in eukaryotic ribosome termination complexes. (**a**) Overall alignment of the PTC from different eukaryotic ribosome structures (6qzp [15] colored in pink is an apo-80S ribosome, 3jag [10] colored in forest green is a termination complex with tRNA and eRF1, and 5a8l [11] colored in grey is the hCMV stalled translation termination complex). Positions of the eRF1 GGQ motif (slate blue), the P-site tRNA (orange) and nascent chain (purple) are shown. (**b-d**) Positions of (**b**) U4501, (**c**) A3887 and (**d**) U4422 aligned within the PTC. Potential steric clashes are highlighted with a red "X" or with labeled atomic distances. (**e**) Cryo-EM density for U4422 and G4499, with U4422 modeled in three different conformations based on the observed density. (**f**) The major conformation of U4422 observed in the cryo-EM density, which makes multiple interactions with the nascent chain and mGln185 of eRF1. Dashed lines indicate hydrogen bonds and spheres represent van der Waals radii. (**g**) Conformation showing U4422 turned outward from the nascent chain, with H-bonds and van der Waals radii as in (**g**). (**h**) Conformation showing U4422 flipped inward to base pair with G4499.

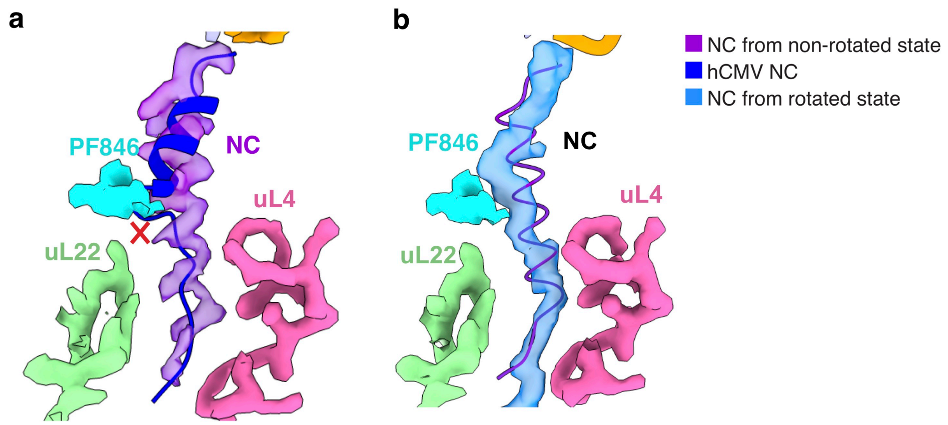

Supplementary Fig. 13 | Comparisons of NCs within ribosomes stalled at termination. (**a**) Superposition of the hCMV nascent chain model (blue, PDB: 5a8l) [11] with the non-rotated CDH1-NPN RNC stalled by PF846. The potential steric clash of the hCMV NC with PF846 is indicated. (**b**) Superposition of the CDH1-NPN NC model from the non-rotated RNC with the cryo-EM density of the rotated RNC.

Supplementary Table 1 | DNA primers used for NC mutagenesis in this study.

Primers for NC mutations	Sequence (5'-3') with mutations in CAPS
P700A- Forward	gcacagGCTgtcgaagcaggattgc
P700A-Reverse	tgcttcgacAGCctgtgccttcctacagacg
V701A- Forward	cacagcctGCTgaagcaggattgcaaattcct
V701A-Reverse	aatcctgcttcAGCaggctgtgccttcctacaga
E702A- Forward	agcctgtcGCTgcaggattgcaaattcctgcc
E702A-Reverse	caatcctgcAGCgacaggctgtgccttcc
L705A-Forward	aagcaggaGCTcaaattcctgccattctgg
L705A-Reverse	aggaatttgAGCtcctgcttcgacaggctg
Q706Q-Forward	caggattgCGAattcctgccattctgggga
Q706Q-Reverse	ggcaggaatCTGcaatcctgcttcgacagg
I707A-Forward	gattgcaaGCTcctgccattctggggattc
I707A-Reverse	aatggcaggAGCttgcaatcctgcttcgac
P708A-Forward	tgcaaattGCTgccattctggggattctt
P708R-Reverse	agaatggcAGCaatttgcaatcctgcttcg
I710A-Forward	ttcctgccGCTctggggattcttggaggaatt
I710A-Reverse	atccccagAGCggcaggaatttgcaatcctgc
L711A-Forward	ctgccattGCTgggattcttggaggaattctt
L711A-Reverse	caagaatcccAGCaatggcaggaatttgcaatcc
G712A-Forward	ccattctgGCTattcttggaggaattcttg
G712A-Reverse	ccaagaatAGCcagaatggcaggaatttgc
I713A-Forward	ttctggggGCTcttggaggaattcttgctttg
I713A-Reverse	cctccaagAGCccccagaatggcaggaatttg
L714N-Forward	tggggattAATggaggaattcttgctttgcta
L714N-Reverse	ttcctccATTaatccccagaatggcaggaa
L714A-Forward	tggggattGCTggaggaattcttgctttgcta
L714A-Reverse	ttcctccAGCaatccccagaatggcaggaa
G715A-Forward	ggattcttGCTggaattcttgctttgctaa
G715A-Reverse	agaattccAGCaagaatccccagaatggca
G716V-Forward	ttcttggaGTCattcttgctttgctaattctg
G716V-Reverse	agcaagaatGACtccaagaatccccagaatg
I717A-Forward	cttggaggaGCTcttgctttgctaattctg
I717A-Reverse	aaagcaagAGCtcctccaagaatccccaga
I717R-Forward	cttggaggaCGGcttgctttgctaattctg
I717R-Reverse	aaagcaagCCGtcctccaagaatccccaga
L718A-Forward	gaggaattGCCgctttgctaattctgattctga
L718A-Reverse	agcaaagcGGCaattcctccaagaatccccaga

A719N-Forward	ggaattcttAATttgctaattctgattctgaat
A719N-Reverse	aattagcaaATTaagaattcctccaagaatc
L720A-Forward	ttcttgctGCTctaattctgattctgaatcca
L720A-Reverse	cagaattagAGCagcaagaattcctccaagaa
L721A-Forward	ttgctttgGCTattctgattctgaatccaaacta
L721A-Reverse	atcagaatAGCcaaagcaagaattcctccaag
I722A-Forward	ctttgctaGCTctgattctgaatccaaactaa
I722A-Reverse	gaatcagAGCtagcaaagcaagaattcctccaa
L723A-Forward	tgctaattGCTattctgaatccaaactaatttcttc
L723A-Reverse	ttcagaatAGCaattagcaaagcaagaattcctcc
I724A-Forward	ctaattctgGCTctgaatccaaactaatttcttc
I724A-Reverse	ggattcagAGCcagaattagcaaagcaagaatt
L725A-Forward	ttctgattGCTaatccaaactaatttcttcggagg
L725A-Reverse	gtttggattAGCaatcagaattagcaaagcaagaa
**Primers for PCSK9 NPN scan**	
**Primers**	**Sequence (5'-3')**
Nluc-F	GTCTTCACACTCGAAGATTTCGTT
PCSK9_27NPN-Reverse	ATCTTCGAGTGTGAAGACGTCCTCgtcctcctgttagtttgg attcgcgggacccag
PCSK9_30NPN-Reverse	ATCTTCGAGTGTGAAGACGTCCTCttagtttggattacggg cgcccgcggga
PCSK9_31NPN-Reverse	ATCTTCGAGTGTGAAGACGTCttagtttggattcgcacgggc gcccgcggga
PCSK9_32NPN-Reverse	ATCTTCGAGTGTGAAGACttagtttggattctgcgcacgggcgc ccgc
PCSK9_33NPN-Reverse	ATCTTCGAGTGTGAAGACttagtttggattctcctgcgcacggg cgccc
PCSK9_34NPN-Reverse	ATCTTCGAGTGTGAAGACttagtttggattgtcctcctgcgcacg ggcgc
PCSK9_35NPN-Reverse	ATCTTCGAGTGTGAAGACttagtttggattCTCgtcctcctgcg cacgggc
PCSK9_36NPN-Reverse	ATCTTCGAGTGTGAAGACttagtttggattGTCCTCgtcctcc tgcgcac
PCSK9_37NPN-Reverse	ATCTTCGAGTGTGAAGACttagtttggattGCCGTCCTCgt cctcctgcgcac

PCSK9_38NPN-Reverse	ATCTTCGAGTGTGAAGACttagtttggattGTCGCCGTCCTCgtcctcctgcgcacgg

Supplementary Table 2 | Primers and gene blocks used for cell line construction.

Sequence motif	Sequence
NPN*	AATCCAAACTAA
GCV*	ggatgtgtctaa
**gBlocks from IDT**	
gBlock1: 5HBB_FLAG_Nluc	CCTCAAACAGACACCATGGACTACAAGGACGA<br>CGACGACAAGATGGTCTTCACACTCGAAGATT<br>TCGTTGGGGACTGGCGACAGACAGCCGGCTA<br>CAACCTGGACCAAGTCCTTGAACAGGGAGGTG<br>TGTCCAGTTTGTTTCAGAATCTCGGGGTGTCC<br>GTAACTCCGATCCAAAGGATTGTCCTGAGCGG<br>TGAAAATGGGCTGAAGATCGACATCCATGTCA<br>TCATCCCGTATGAAGGTCTGAGCGGCGACCAA<br>ATGGGCCAGATCGAAAAAATTTTTAAGGTGGT<br>GTACCCTGTGGATGATCATCACTTTAAGGTGAT<br>CCTGCACTATGGCACACTGGTAATCGACGGGG<br>TTACGCCGAACATGATCGACTATTTCGGACGG<br>CCGTATGAAGGCATCGCCGTGTTCGACGGCAA<br>AAAGATCACTGTAACAGGGACCCTGTGGAACG<br>GCAACAAAATTATCGACGAGCGCCTGATCAAC<br>CCCGACGGCTCCCTGCTGTTCCGAGTAACCAT<br>CAACGGAGTGACCGGCTGGCGGCTGTGCGAA<br>CGCATTCTGGCG

gBlock2: CDH1-NPN*_3'UTR_bGH	TCTGATGTGAATGACAACGCCCCCATACCAGA ACCTCGAACTATATTCTTCTGTGAGAGGAATCC AAAGCCTCAGGTCATAAACATCATTGATGCAGA CCTTCCTCCCAATACATCTCCCTTCACAGCAGA ACTAACACACGGGGCGAGTGCCAACTGGACCA TTCAGTACAACGACCCAACCCAAGAATCTATCA TTTTGAAGCCAAAGATGGCCTTAGAGGTGGGT GACTACAAAATCAATCTCAAGCTCATGGATAAC CAGAATAAAGACCAAGTGACCACCTTAGAGGT CAGCGTGTGTGACTGTGAAGGGGCCGCTGGC GTCTGTAGGAAGGCACAGCCTGTCGAAGCAG GATTGCAAATTCCTGCCATTCTGGGGATTCTTG GAGGAATTCTTGCTTTGCTAATTCTGATTCTGct gctcttgctgTTTCTTCGGAGGAGAGCGGTGGTCAA AGAGCCCTTACTGCCCCCAGAGGATGACACCC GGGACgggactcgagagaggcgggccccagacccatgtgctg ggaaatgcagaaatcacgttgctaCTCGAGcgactgtgccttcta gttgccagccatctgttgtttgcccctcccccgtgccttccttgaccctg gaaggtgccactcccactgtcctttcctaataaaatgaggaaattgca tcgcattgtctgagtaggtgtcattctattctggggggtggggtggggc aggacagcaagggggaggattgggaagacaatagcaggcatgc tggggatgcggtgggctctatggCTCGAG
**For NPN* stable cell line**	
**Primers**	**Sequence (5'-3')**
1. Use gBlock1 as template to make fragment: FLAG (overlap 5'UTR of HBB) and NLuc	
Forward	CCTCAAACAGACACCCCATGGACTACAAGGAC GACGA
Reverse	CGCCAGAATGCGTTCGCACA
2. Use 1 as template to make fragment: HBB_FLAG_Nluc	
Forward	ACATTTGCTTCTGACACAACTGTGTTCACTAGC AACCTCAAACAGACACCATGGACT
Reverse	CGCCAGAATGCGTTCGCACA
3. Use gBlock2 as template to make fragment: Nluc_CDH1_bGH	
Nluc_CDH1_F	CTGTGCGAACGCATTCTGGCGTCTGATGTGAA TGACAACGCC
bGH_R	ATTGTCGACTCGAGCCATAGAGCCCAC
4. Fuse Fragments 2 and 3	

5. Use 4 as template to make fragment from 5HBB to bGH, with overlap with EF1	
EF1α_5HBB_F	TTTCAGGTGTCGTGAGTCGAACATTTGCTTCTGACACAACTG
bGH_WPRE_R	ATCCAGAGGTTGATTGTCGACTCGAGCCATAGAGCCCAC
6. Cut CD813 vector with Sal1	
7. Ligate fragment 5 with CD813 vector	
**For GCV* stable cell line**	
**Primers**	**Sequence (5'-3')**
Use NPN* construct as the template	
1. Fragment: 5HBB (overlap with EF1α)_FLAG_Nluc_CDH1_GCV	
EF1α_5HBB_F	TTTCAGGTGTCGTGAGTCGAACATTTGCTTCTGACACAACTG
Lenti_GCV_R	CACCGCTCTCCTCCGAAGAAAttagacacatccCAGAATCAGAATTAGCAA
2.Fragment: GCV_3UTR_bGH	
CDH1-730F	TTTCTTCGGAGGAGAGCGGTGGTCAA
bGH_WPRE_R	ATCCAGAGGTTGATTGTCGACTCGAGCCATAGAGCCCAC
Fuse fragment 1 and 2 to CD813 vector	
**Make construct for Q706A stable cell line**	
**Primers**	**Sequence (5'-3')**
Use NPN* construct as the template	
1. Fragment: 5HBB (overlap with EF1α)_FLAG_Nluc_CDH1_Q706A	
EF1α_5HBB_F	TTTCAGGTGTCGTGAGTCGAACATTTGCTTCTGACACAACTG

Lenti_Q706A_R	CAGGAATagcCAATCCTGCTTCGACAGGCTG
2.Fragment: 706A_3UTR_bGH	
Lenti_Q706A_F	GCAGGATTGGCTATTCCTGCCATTCTGGGG
bGH_WPRE_R	ATCCAGAGGTTGATTGTCGACTCGAGCCATAG AGCCCAC
Fuse fragment 1 and 2 to CD813 vector	
**Make construct for L714A stable cell line**	
**Primers**	**Sequence (5'-3')**
Use NPN* construct as the template	
1. Fragment: 5HBB (overlap with EF1α)_FLAG_Nluc_CDH1_Q706A	
EF1α_5HBB_F	TTTCAGGTGTCGTGAGTCGAACATTTGCTTCTG ACACAACTG
Lenti_L714A_R	ttcctccAGCaatccccagaatggcaggaa
2.Fragment: 706A_3UTR_bGH	
Lenti_L714A_F	tggggattGCTggaggaattcttgctttgcta
bGH_WPRE_R	ATCCAGAGGTTGATTGTCGACTCGAGCCATAG AGCCCAC
Fuse fragment 1 and 2 to CD813 vector	

Supplementary data set 1 | Source data for the bar graphs presented in the main text.

Supplementary data set 2 | The uncropped images for gels shown in Fig. 3e, Supplementary Fig. 10e and g.

Supplementary data set 3 | The uncropped images for gels shown in Fig. 2c, Supplementary Fig. 7c and 8b-c.

Supplementary data set 4 | The uncropped images for gels shown in Supplementary Fig. 8c.

Supplementary data set 5 | The uncropped images for gels shown in Supplementary Fig. 10f and Fig. 3e.
