# Supplementary material for: Selective inhibition of human translation termination by a drug-like compound
Source: Nat Commun. 2020 Oct 2;11:4941. doi: 10.1038/s41467-020-18765-2 (PMC7532171; doi:10.1038/s41467-020-18765-2)
Supplement: Supplementary file 4 — Source Data [file 41467_2020_18765_MOESM4_ESM.zip › Li_etal_Supplementary Data Set 2.pdf]

Li_etal_Supplementary Data Set 2 (Fig.3e——mutants that affect the stalling; and Extended Data Fig. 10e,g)

Fig.3e

718-727 (NPN is the control, not used in the final figure)

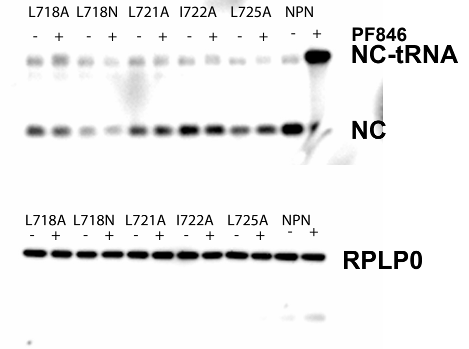

Supplementary Fig.10e

714-717 FLAG

(NPN is the control, not used in the final figure)

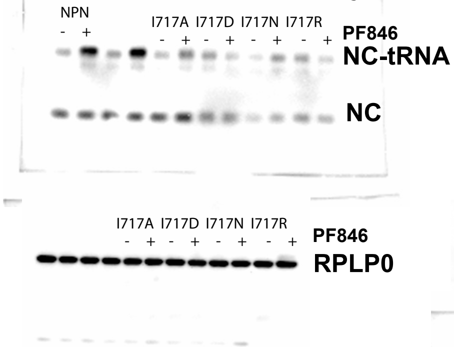

Note: The same control sample was run on all the gels.

Supplementary Fig.10e

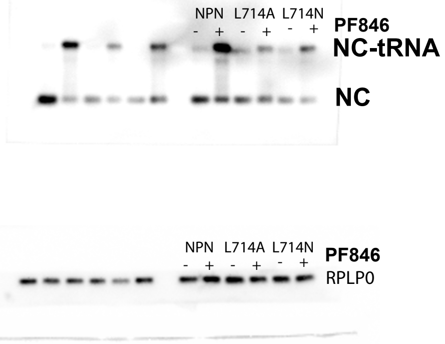

Supplementary Fig.10g

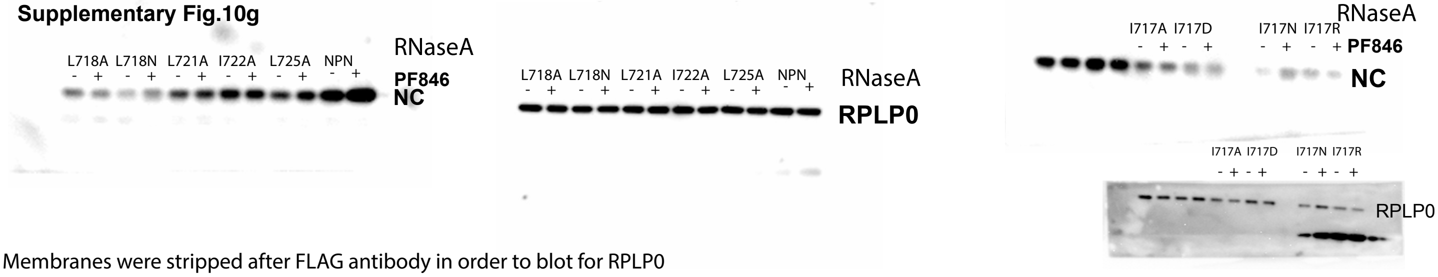

Membranes were stripped after FLAG antibody in order to blot for RPLP0
