# Supplementary material for: Selective inhibition of human translation termination by a drug-like compound
Source: Nat Commun. 2020 Oct 2;11:4941. doi: 10.1038/s41467-020-18765-2 (PMC7532171; doi:10.1038/s41467-020-18765-2)
Supplement: Supplementary file 4 — Source Data [file 41467_2020_18765_MOESM4_ESM.zip › Li_etal_Supplementary Data Set 3.pdf]

Li_etal_Supplementary Data Set 3 (Fig. 2c, Supplementary Fig. 7c and 8b)

Supplementary Fig. 7c

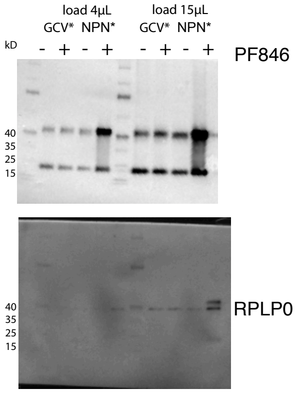

Supplementary Fig. 8b

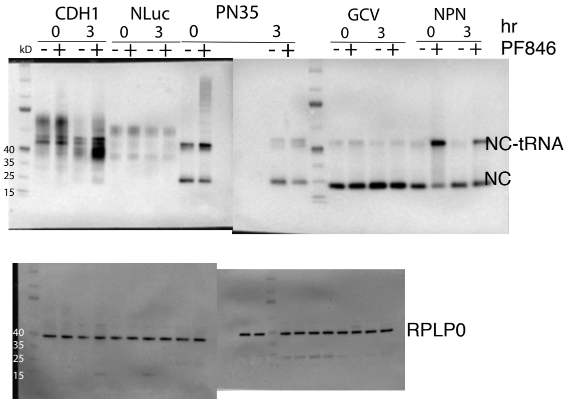

Fig. 2c

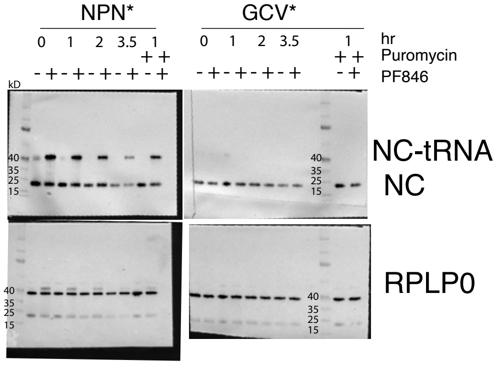

Membranes were stripped after FLAG antibody in order to blot for RPLP0
