# Supplementary material for: Selective inhibition of human translation termination by a drug-like compound
Source: Nat Commun. 2020 Oct 2;11:4941. doi: 10.1038/s41467-020-18765-2 (PMC7532171; doi:10.1038/s41467-020-18765-2)
Supplement: Supplementary file 4 — Source Data [file 41467_2020_18765_MOESM4_ESM.zip › Li_etal_Supplementary Data Set 5.pdf]

Li_etal_Supplementary Data Set 5 (Supplementary Fig. 10f and Fig.3e——mutants that don't affect the stalling)

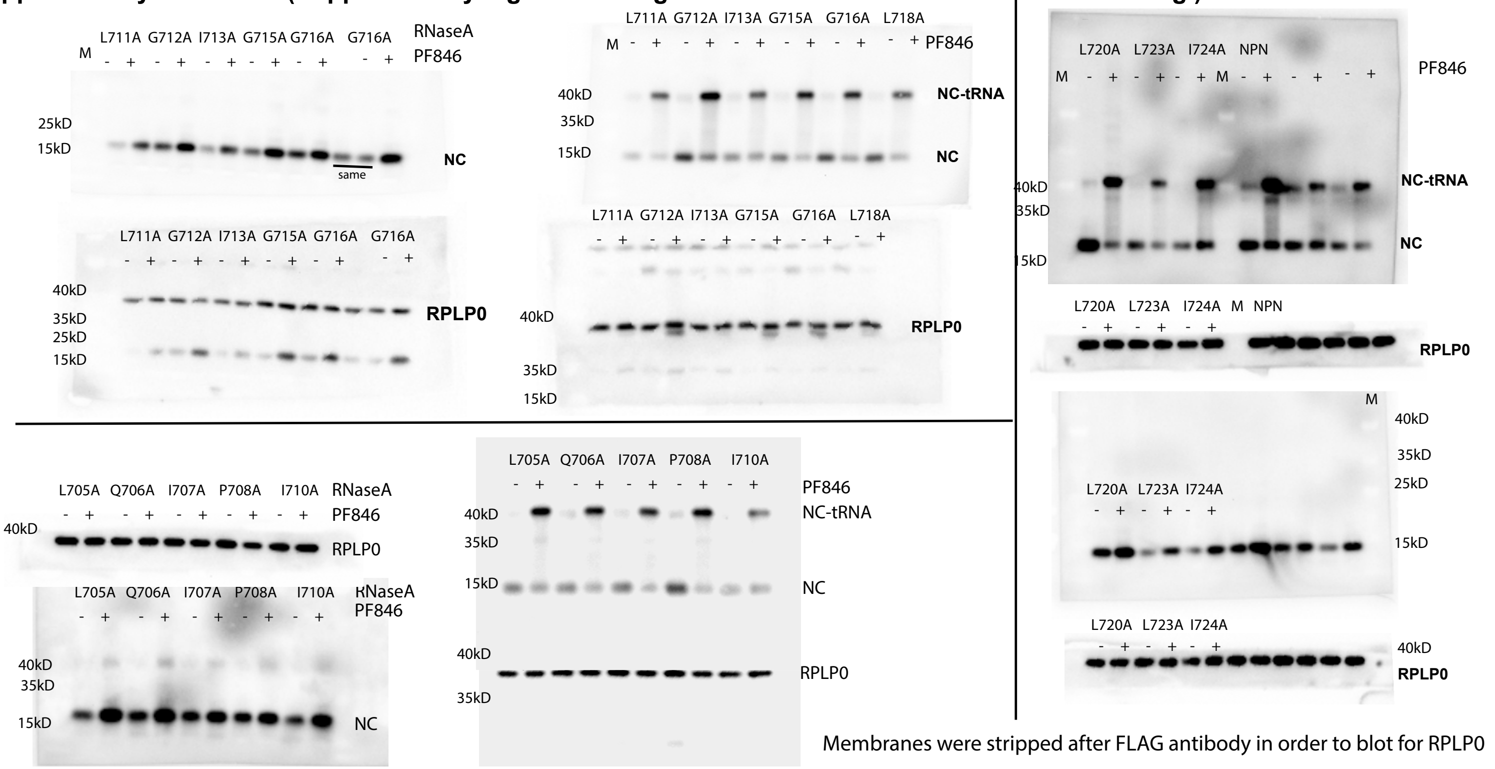

Membranes were stripped after FLAG antibody in order to blot for RPLP0
