# Supplementary material for: Selective inhibition of human translation termination by a drug-like compound
Source: Nat Commun. 2020 Oct 2;11:4941. doi: 10.1038/s41467-020-18765-2 (PMC7532171; doi:10.1038/s41467-020-18765-2)
Supplement: Supplementary file 2 — Peer Review [file 41467_2020_18765_MOESM2_ESM.pdf]

Reviewer #1 (Remarks to the Author):

In this manuscript, Li et al. expand on their previous work of the effects of a small molecule, PF846, on translation in mammalian cells. In the previous study, they have characterized the PF846-induced translation elongation arrest at few specific ORFs. In that work they also have found that PF846 stalls the ribosome at the stop codon that follows the Asn-Pro-Asn coding sequence. In the current work they have analyzed the structure of the mammalian ribosome stalled by PF846 at the stop codon of the NBPBN-stop sequence. They describe the conformation of the stalling nascent protein chain (NC) and its contacts with the exit tunnel. They also found that the conformation of several nucleotide residues in the peptidyl transferase center (PTC) and the exit tunnel are incompatible with eRF1-catalyzed peptidyl-tRNA hydrolysis. They also suggest that the nascent chain is retained in the ribosome after the peptidyl-tRNA ester bind is hydrolyzed.

Critique:
This is an interesting study that deepens the understanding of the mode of action of a highly-selective inhibitor of eukaryotic translation, PF846. The study is generally well done and provides interesting insights into the structure of the terminating ribosome and the possible effects of the nascent chain and exit tunnel-bound small molecule on peptide release. Thereis, however one important aspect of the story, or rather the claim, that lacks any experimental support. Specifically, I remained completely unconvinced that the nascent chain is retained in the ribosome AFTER peptidyl-tRNA hydrolysis. This claim is first made on p.6. (Fig. 2a, and Extended Data Fig. 6 b,c). This is a really toll claim that requires strong evidence. However, the ONLY evidence in favor of it is a break in the cryo-EM density between the P-site tRNA and nascent chain (NC). Because authors do not comment that either NC or tRNA have moved I assume that the respective atoms of the tRNA and NC are still at the bonding distance. Given the resolution of the structure (very decent, but yet…), I am not sure whether one can really conclude that the ester bond is not there. The authors further claim that the in vitro biochemical evidence supports their assertion, but it does not! They write: "Consistent with the break in the density, the peptidyl tRNA is slowly hydrolyzed… in RNC purified from in vitro translation reactions". In fact, this observation argues just the opposite: that it is peptidyl-tRNA hydrolysis that is inhibited rather than that the nascent chain is retained after the hydrolysis has occurred. In the ribosomes purified after in vitro translation, most of peptidyl-tRNA is unhydrolyzed, whereas the amount of apparently ribosome-associated hydrolyzed NC is similar between the samples with and without PF846 (Extended Data Fig. 6 b). I truly believe that without any strong experimental support for the retention of the NC in the ribosome after peptidyl-tRNA hydrolysis, the observation of a break in cryo-EM density deserves one sentence in the Discussion rather than being as a basis for one of the main claims of the paper.

Other smaller issues:

p. 7. and Fig. 3b: The discussion of the role of the C-terminal residue (N728) is confusing. If I understood authors correctly, all amino acids but Tyr and Trp support PF846- mediated arrest at the stop codon of the NPN-Stop sequence. What is then the meaning of the statement that "N728 projects towards a pocket formed by 28S rRNA residues G3886-C3888 that leaves room for larger amino acid side chains". Do they mean that the pocket is big enough for any side chain but Tyr and Trp? Or is it big enough for any amino acid? What is the relevance of this pocket for the arrest mechanism? Also, what about Arg and Lys which have long-side chains: will they fit in the pocket?

p.11. I suspect that ref. 30 should be Polacek et al. (2003) Mol Cell.

p.11. The discussion of the link between the role of A3887 -A4419 base pair in macrolide-dependent stalling in bacteria and PF846-dependent stalling in human cells was presented in a rather convoluted way and would benefit from re-writing. Furthermore, in my recollection, the role of this interaction in macrolide-dependent stalling was initially shown by Vazquez-Laslop et al. (EMBO J, 2010).

p.29. Indicate the volume of the sucrose cushion.

p.29. The design of the in vitro experiment and in particular, the purpose of RNase treatment is unclear. What was the rational for treating RNC with RNase A? Would not the ribosome-bound peptidyl-tRNA be protected from RNase? Furthermore, how treating the sample at 55°C in Laemmli buffer help to visualize 'tRNA-bound stalled nascent chains"? Would not peptidyl-tRNA be hydrolyzed under these conditions?

Extended Data Fig. 5, panels a and b. Are the panels a and b switched in the figure? Anyway, it feels that just one of them would be enough to convey the message.

Extended Data Fig. 6. The experimental setup is unclear. According to the methods section, the RNC complex was pelleted though the sucrose cushion. If so, was the hydrolyzed NC (middle section in panel b) retained in the ribosomes not exposed to PF846 (the '-' samples)? The experiment also lacks the positive control: what should be the effect of PF846 on kinetics of hydrolysis of the RNC that is not conducive to the PF846 action?

Extended Data Fig. 11a. It is unclear what message the authors are trying to convey with showing the alignment of three "eukaryotic ribosome structures" moreover that only the PDB IDs are shown without any reference to what these structures represent.

Reviewer #2 (Remarks to the Author):

The manuscript follows previous publications from the same group investigating the drug-like compound PF846 that was shown to selectively inhibit synthesis of specific proteins. Their previous studies highlighted how it inhibits elongation and here they invetigate how it can also inhibit termination. In general, the results are exciting however the manuscript suffers from its brevity, Furthermore, the data for the main conclusion of the paper that PF846 has a unique mechanism of action to allow termination to occur but trap the NC on the ribosome after hydrolysis, does not in my opinion, fully support the conclusion. I believe that it inhibits termination, but I am not convinced that it is after peptide bond formation based on the presented data.

Major comments
1. Fig 2. It needs to be made clear whether the cryo-EM map densities are segmented or not. I would presume that they segnmented for the colored ones and not for the inset. However, this needs to be always stated clearly. Moreover, different thresholds need to be shown for the unsegmented map to see if density does in fact connect the A76 with the NC at lower thresholds. Either way I don't find this evidence to make the statement on page 6 that it indicates hydrolysis of the ester bond without supporting biochemistry, which has not been performed up to this point in the paper.
2. In Ed Fig. 6, the authors show that the NC-tRNA becomes "slowly hydrolyzed on the hour timescale in RNCS purified from in vitro translation reactions" – the authors need to add the important point "in the presence of PF846". The RNC is rapidly hydrolyzed even on ice in the absence of the drug! More importantly however I fail to understand how these findings fit with their results…one would presume that isolation of an RNC for cryo-EM studies is not done at room temperature but on ice or 4deg, Moreover, it would seem most relevant to actually perform the experiments on the sample that was used for the cryo-EM study (or at least treated the same way) to see if the NC-tRNA is intact?
3. I do not see the pivotal experiment showing that the NC remains on the ribosome dependent on the presence of the drug. This could be done by monitoring association of the NC with the ribosome fraction after pelleting assays, in presence and absence of the drug. Controls would include puromycin to show that the NC is released when the drug is not present and when the drug is added, puromycin should still lead to loss of the peptidyl-tRNA band, but the NC should still be detected in the ribosome fraction in a drug-dependent fashion. In fact, dose-dependence of the drug would be the most

convincing.
4. The authors discuss their results within the context of termination and seem to have forgotten that there is an elongation effect, for example, for ED Fig. 1 and ED Fig. 6, it seems inappropriate to state here inhibition of translation termination since this assay does not monitor specifically termination. In fact, the structural analysis shows that it's a mixture of inhibition of termination and elongation complexes. How does this influence the interpretation of the other results in the paper e.g. Fig. 3e and ED Fig. 8-9. Might one argue based on particle numbers from ED Fig. 2 that termination if 66% and elongation is 33% of the complex?
5. Lastly, assuming that the authors are correct and that the inhibition by PF846 is post-hydrolysis of the NC by eRF1, then it is unclear to me which step is then inhibited? I-i.e. is it eRF1 dissociation? How does PF846 prevent eRF1 dissociation then? A simpler model would be that eRF1 remains because it cannot hydrolyze the ester linker.

Minor comments
1. The manuscript suffers from its brevity, presumably having fallen down from being sent to another journal with tighter space restrictions. For example, the entire paper is based on observations that PF846 inhibits not only elongation but also termination as reported in reference 4 but no details are given. Maybe the evidence should be summerized in the introduction too? Why were sequences NPN* added to Cadherin-1? The rationale for the initial results is lacking in this manuscript with the expectation that the reader should first read reference 4. Is it at a particular stop codon? How did it come about that GCV is the selected control sequence in reference 4? Moreover, the discussion is incomplete with reference to other stalling mechanisms and surprisingly, does not even discuss the numerous studies reporting antimicrobial peptides that block termination after the release factor has hydrolyzed the ester linkage.
2. Page 3: There lacks clarity in the last sentence of the first paragraph. Treatment of cells with PF846 inhibits luciferase fused to CDH-NPN*…this is seen in Fig. 1a however because it's a log scale without many points on the x-axis its hard to understand how the 3uM IC50 was calculated. That CDH1-GCV is not inhibited is not true, there is also inhibition seen in Fig. 1a, just not as dramatic. To help the reader, perhaps the authors should consider putting the (Fig. 1a) at the end of this sentence rather after the "consistent with in vitro translation results". One assumes then that the in vitro is ED Fig. 1...actually Fig. 1b…but this also seems to be an "in cell assay" as indicated by the title. So if Fig. 1b is also in cells, where is the in vitro data? Maybe the authors expect the reader to understand that this is in reference 4? Also with regard to ED Fig. 1b, the small symbols in the key of the figure cannot be seen clearly…perhaps just use black and white squares as indicated in the legend?
3. On page 6 (para 2, line 9-10), I find it inappropriate to cite Fig. 2a but rather only ED Fig. 6 at this point. Especially since I don't find anything in the biochemistry consistent with the break in the density…see major points above.
4. The use of PCSK9 is confusing. The authors write that it is "unlikely to form a compact a-helical geometry" but then show Phyre predictions of a-helices? Its also unclear why the authors now change the format of the presentation to treat all samples with RNase A and do not use the representation as in Fig. 3e. Also using a positive and negative control would be good.
5. In Fig. 3e, it is unfortunate that every mutation basically abolishes the stalling. The reader has to look to ED Fig. 9 to be convinced that there are amino acid position specific effects. Perhaps it would be good to have a few negative control mutations in Fig. 3e too.
6. If an intact NPN motif is needed, how many proteins have intact NPN before a stop codon in the human genome? Obviously, more than an NPN motif is needed, so how many of these NPN* proteins are affected by PF846? Are there proteins also affected during elongation too? i.e. are these activities separable? Is there a secondary structure feature requirement before the NPN motif? Do mutations that break the helix also lead to loss of stalling?

REVIEWER COMMENTS

Reviewer #1 (Remarks to the Author):

In this manuscript, Li et al. expand on their previous work of the effects of a small molecule, PF846, on translation in mammalian cells. In the previous study, they have characterized the PF846-induced translation elongation arrest at few specific ORFs. In that work they also have found that PF846 stalls the ribosome at the stop codon that follows the Asn-Pro-Asn coding sequence. In the current work they have analyzed the structure of the mammalian ribosome stalled by PF846 at the stop codon of the NPN-stop sequence. They describe the conformation of the stalling nascent protein chain (NC) and its contacts with the exit tunnel. They also found that the conformation of several nucleotide residues in the peptidyl transferase center (PTC) and the exit tunnel are incompatible with eRF1-catalyzed peptidyl-tRNA hydrolysis. They also suggest that the nascent chain is retained in the ribosome after the peptidyl-tRNA ester bond is hydrolyzed.

Critique:

This is an interesting study that deepens the understanding of the mode of action of a highly-selective inhibitor of eukaryotic translation, PF846. The study is generally well done and provides interesting insights into the structure of the terminating ribosome and the possible effects of the nascent chain and exit tunnel-bound small molecule on peptide release. There is, however one important aspect of the story, or rather the claim, that lacks any experimental support. Specifically, I remained completely unconvinced that the nascent chain is retained in the ribosome AFTER peptidyl-tRNA hydrolysis. This claim is first made on p.6. (Fig. 2a, and Extended Data Fig. 6 b,c). This is a really tall claim that requires strong evidence. However, the ONLY evidence in favor of it is a break in the cryo-EM density between the P-site tRNA and nascent chain (NC). Because authors do not comment that either NC or tRNA have moved I assume that the

respective atoms of the tRNA and NC are still at the bonding distance. Given the resolution of the structure (very decent, but yet…), I am not sure whether one can really conclude that the ester bond is not there. The authors further claim that the in vitro biochemical evidence supports their assertion, but it does not! They write: "Consistent with the break in the density, the peptidyl tRNA is slowly hydrolyzed… in RNC purified from in vitro translation reactions". In fact, this observation argues just the opposite: that it is peptidyl-tRNA hydrolysis that is inhibited rather than that the nascent chain is retained after the hydrolysis has occurred. In the ribosomes purified after in vitro translation, most of peptidyl-tRNA is unhydrolyzed, whereas the amount of apparently ribosome-associated hydrolyzed NC is similar between the samples with and without PF846 (Extended Data Fig. 6 b). I truly believe that without any strong experimental support for the retention of the NC in the ribosome after peptidyl-tRNA hydrolysis, the observation of a break in cryo-EM density deserves one sentence in the Discussion rather than being as a basis for one of the main claims of the paper.

We thank the reviewer for their interest in the present manuscript, and for the critique of our model on how PF846 inhibits translation termination. Both reviewers raised similar concerns about the trapping of the released NC in the ribosome exit tunnel by PF846, so we will address those here. First, we will clarify the model we originally proposed for how PF846 inhibits translation termination on the CDH-NPN* sequence. Then, we will review our data and present new results in support of a revised model. We thank the reviewers for independently raising their concern, as it has helped us clarify the model, both in our description and through new experiments.

First, based on the cryo-EM and biochemical data, we proposed the following model for PF846-mediated inhibition of translation termination in the original manuscript. PF846 initially slows down translation of the NC as the ribosome approaches the stop codon. At the stop codon, PF846 then prevents rapid hydrolysis of the NC from the P-site tRNA by eRF1. Finally, even after the NC release, strong interactions with PF846 and the exit tunnel can still arrest the NC in the ribosome exit tunnel. In summary, the original model

was that PF846 affects translation of the CDH1-NPN* NC at three steps: elongation, peptidyl-tRNA hydrolysis, and NC release from the ribosome exit tunnel.

Based on new experiments described below, we have modified our model to indicate that PF846 likely inhibits translation of the CDH1-NPN* sequence by the first two steps: slowing down elongation near the stop codon, and inhibiting rapid hydrolysis of the NC from the P-site tRNA. We now agree with the reviewers that PF846 by itself likely does not trap the nascent chain in the ribosome exit tunnel. As described below, some NCs are inherently "sticky" and can remain trapped in the ribosome exit tunnel even in the absence of PF846, at least *in vitro*.

Here, we will summarize the original and new data supporting the new model, beginning with PF846 effects on elongation near the stop codon. First, by cryo-EM we observe about ⅓ of the particles isolated with the NC in the rotated state (**Fig. 1b and ED Fig. 2a**). The resulting structure is highly similar to the stalled elongation complexes described in (Li *et al*., 2019, *NSMB*). In addition to being in the rotated state, the density for the mRNA and tRNA anticodon stem-loops have similar levels to that of the nearby 18S rRNA, indicating the A site and P site of the 40S subunit are fully occupied by bound tRNA. Also like the prior structures, the density for the mRNA codon and tRNA anticodon bases is blurred, and can't be fit cleanly with either pyrimidines or purines (**ED Fig. 3a-c**). This is reminiscent of the elongation stalling observed over multiple codons by ribosome profiling in the original CDH1 sequence context (Lintner *et al.*, 2017, *PLOS Biol.*). Notably, we also observe that the CCA end of the A/P tRNA is somewhat disordered, and the density of the NC cannot be fit with a defined amino acid sequence. This is also similar to the stalled elongation complexes in the 2019 *NSMB* paper, as is the overall conformation of the NC in the ribosome exit tunnel (**Fig. 2a and ED Fig. 13b**). Given that the CDH1-NPN* sequence is nearly identical to the original CDH1 sequence apart from the 4 amino acids proximal to the predicted stall site, and the structural similarity of this class of particles to the PF846-stalled CDH1-RNC

complex, we think it is reasonable to propose that PF846 slows elongation of the CDH1-NPN* sequence near the stop codon.

We also showed that PF846 inhibits translation termination by blocking NC hydrolysis from the P-site tRNA, both structurally and biochemically. By cryo-EM, we observe about ⅔ of the particles in the non-rotated state with the stop codon in the ribosomal A site, and eRF1 bound to the ribosome (**Fig. 1c and Fig. 2b**). This complex also has P-site tRNA and the NC in the ribosome exit tunnel (**Fig. 2b and Fig. 3a**). Interestingly, in the PTC, U4501 is flipped away due to the potential steric clash with the NC which likely suppresses PTC activity (**Fig. 4b and e**). Biochemically, we observe a substantial amount of NC-tRNA trapped in the ribosome in a PF846-dependent and NC sequence-dependent manner (**Fig. 3e**, **ED Fig. 7c, ED Fig. 8b-c,** and **ED Fig. 10e-g**). Interestingly, PF846 has no effect on the CDH1-GCV* NC or a nanoluciferase NC, which we now show in **ED Fig. 7-8**. We also have repeated translation reactions with one of the PCSK9-NPN* termination NCs and find PF846 only weakly inhibits NC hydrolysis from the P-site tRNA in this case (**ED Fig. 8b**). From these structural and biochemical experiments, we show that PF846 can selectively inhibit hydrolysis of the NC from P-site tRNA by eRF1.

The most controversial aspect of our original model to both reviewers is that PF846 can trap the NC in the ribosome exit tunnel even after it is hydrolyzed from the P-site tRNA CCA end. From new biochemical data and reexamining our previous data, we now agree with the reviewers. First, in the structure of the stalled termination complex, the cryo-EM density for the NC-tRNA ester bond is weak, but not completely absent at lower contour levels (**ED Fig. 7b**). Biochemically, we now have ample evidence that some NC sequences are trapped on ribosomes *in vitro*, after ribosome pelleting (**ED Fig. 8**). In translation reactions with either the CDH1-NPN* or CDH1-GCV* sequences (the latter not a target of PF846), NC hydrolyzed from the P-site tRNA copurified with pelleted ribosomes (**ED Fig. 7c and 8**). This is true both in the presence and absence of PF846 (**ED Fig. 8b-c**) and is also true for all of the CDH1-NPN* mutant sequences (**Fig.**

3e and ED Fig. 10). Notably, PCSK9-NPN* but not Nanoluc NC released from P-site tRNA can also be detected in pelleted ribosomes even without PF846 present (**ED Fig. 8b**). We also repeated the FLAG-tag based RNC purification as used for cryo-EM sample preparation, and found both NC-tRNA and NC hydrolyzed from P-site tRNA in the sample (**ED Fig. 7c**). In summary, we think the cryo-EM density reflects a mixture of NC-tRNA and NC hydrolyzed from P-site tRNA. However, the presence of the hydrolyzed NC is not due to trapping by PF846, but is an inherent biochemical property of the NC itself. Importantly, we reinterpret our cell-based experiments with CDH1-NPN* and CDH1-GCV* (**Fig. 1a and ED Fig. 10h**) in light of these new findings. We think that PF846 inhibits NC-tRNA hydrolysis by eRF1, and in cells, the remaining NC after hydrolysis is removed from ribosomes. Thus the NC we see released from P-site tRNA but trapped in the ribosome exit tunnel *in vitro* is not likely to represent the situation in cells. We have extensively rewritten the manuscript to reflect our new model and these new findings.

Other smaller issues:

p. 7. and Fig. 3b: The discussion of the role of the C-terminal residue (N728) is confusing. If I understood authors correctly, all amino acids but Tyr and Trp support PF846- mediated arrest at the stop codon of the NPN-Stop sequence. What is then the meaning of the statement that “N728 projects towards a pocket formed by 28S rRNA residues G3886-C3888 that leaves room for larger amino acid side chains”. Do they mean that the pocket is big enough for any side chain but Tyr and Trp? Or is it big enough for any amino acid? What is the relevance of this pocket for the arrest mechanism? Also, what about Arg and Lys which have long-side chains: will they fit in the pocket?

In the previous study, we identified the NPN* motif from the randomized mRNA library (Li *et al*., 2019, *NSMB*). From the mRNA library results, we observed the 728 position of

the nascent chain can accommodate most amino acids with the exception of Trp and Tyr. We thereby analyzed the structure and found the 28S rRNA residues G3886-C3888 form a pocket which can accommodate any amino acid at position 728 except Trp and Tyr. This includes Arg and Lys. We have new **ED Fig. 9** showing models for Arg and Lys in this pocket.

p.11. I suspect that ref. 30 should be Polacek et al. (2003) Mol Cell.

Thank you for the suggestion. We have added this reference here in the revised manuscript: "In bacteria two universally conserved rRNA nucleotides, Ec A2602 and Ec U2585 (A4518 and U4501 in human 28S rRNA, respectively) are required for peptide release (Koch et al. 2017; Youngman et al. 2004; Polacek et al. 2003)."

p.11. The discussion of the link between the role of A3887 -A4419 base pair in macrolide-dependent stalling in bacteria and PF846-dependent stalling in human cells was presented in a rather convoluted way and would benefit from re-writing. Furthermore, in my recollection, the role of this interaction in macrolide-dependent stalling was initially shown by Vazquez-Laslop et al. (EMBO J, 2010).

Thank you for pointing this out. We now cite this reference and have rephrased this section in the revised manuscript: "In the ribosome exit tunnel, the universally conserved nucleotides A3887 and A4419 (*Ec* A2062 and *Ec* A2503, respectively) form a non-canonical A-A base pair (**Fig. 4f**), which is essential in macrolide-dependent ribosome stalling (**Fig. 5c**) (Koch et al. 2017; Arenz et al. 2014; Vázquez-Laslop et al. 2010). This base pair makes multiple interactions with NC residues critical for PF846-stalled translation termination (**Fig. 3b, d and g**)"

p.29. Indicate the volume of the sucrose cushion.

Thank you for the careful reading. We added the volume of the sucrose cushion in the methods: “The supernatant was applied to a 50% sucrose cushion (260 μL) prepared with cushion buffer.”

p.29. The design of the in vitro experiment and in particular, the purpose of RNase treatment is unclear. What was the rationale for treating RNC with RNase A? Would not the ribosome-bound peptidyl-tRNA be protected from RNase?

We thank the reviewer for raising this question. RNase A is a small mainly nonspecific ribonuclease and is not blocked from cleaving tRNAs in the ribosome. It is commonly used in RNC studies to cleave the NC from NC-tRNA, for example in (Tesina, 2019, *NSMB*).

In our manuscript, we used RNase A treatment to release tRNA-bound NC, giving a single population of NC species to aid quantification of Western blots. In the revised manuscript, we include all the NC mutagenesis related RNC samples with RNase A treatment in revised **ED Fig. 10f-g**.

Furthermore, how treating the sample at 55°C in the Laemmli buffer help to visualize ‘tRNA-bound stalled nascent chains”? Would not peptidyl-tRNA be hydrolyzed under these conditions?

Treating the sample at 55 °C in Laemmli buffer (pH 6.8) and subsequent Bis-Tris NuPAGE gel (pH 6.8) allowed us to visualize NC-tRNA. Tesina *et al.* (2019, *NSMB*) also used this protocol to visualize the NC-tRNA by Western blotting. We contacted the first author directly for advice on this technique. We have cited their paper as a reference and now acknowledge him in the Acknowledgements.

Extended Data Fig. 5, panels a and b. Are the panels a and b switched in the figure? Anyway, it feels that just one of them would be enough to convey the message.

We apologize for the confusing panels. The previous **ED Fig.5** is now **ED Fig. 4** in the revised manuscript. Panel **a** shows the RNC model in surface mode in ChimeraX. Panel **b** is the real density as you can see the "foot" of 40S subunit is discontinuous. We clarified this in the figure legend "(**a**) Comparison of the atomic model (shown with surface in ChimeraX (66) (Goddard et al. 2018)) of the 40S subunit in the rotated state (light cyan) with the non-rotated state (dark purple)."

Extended Data Fig. 6. The experimental setup is unclear. According to the methods section, the RNC complex was pelleted through the sucrose cushion. If so, was the hydrolyzed NC (middle section in panel b) retained in the ribosomes not exposed to PF846 (the '-' samples)? The experiment also lacks the positive control: what should be the effect of PF846 on kinetics of hydrolysis of the RNC that is not conducive to the PF846 action?

Thank you for raising these points. We have added new data to **ED Fig. 7-8**, combining it with the previous **ED Fig. 8**, and extensively rewritten the sections of the manuscript that refer to it. We hope the rationale for these experiments and the results are now clear.

Extended Data Fig. 11a. It is unclear what message the authors are trying to convey with showing the alignment of three "eukaryotic ribosome structures" moreover that only the PDB IDs are shown without any reference to what these structures represent.

The previous **ED Fig. 11** is now **ED Fig.12** in the revised manuscript**. Extended Data Fig. 12a** is an overall view for panels **b-d**. It shows the PTC and exit tunnel nucleotide A3887 from three representative eukaryotic ribosomes structures. We think the confusion is due to the lack of descriptions of each model cited in the figure. In the revised manuscript, we added the citation of **Extended Data Fig. 12a** in this sentence: "However, nucleotide U4501 (*Ec* U2585) is rotated by 90° away from the PTC, to avoid a steric clash with P727 in the nascent chain (**Fig. 4b and e, Extended Data Fig. 12a-b**)." We also added this sentence in the figure legend to clarify what the structures represent: "6qzp [5] colored in pink is an apo-80S ribosome, 3jag [6] colored in forest green is a termination complex with tRNA and eRF1, and 5a8l [3] colored in grey is the hCMV-stalled translation termination complex."

Reviewer #2 (Remarks to the Author):

The manuscript follows previous publications from the same group investigating the drug-like compound PF846 that was shown to selectively inhibit synthesis of specific proteins. Their previous studies highlighted how it inhibits elongation and here they invetigate how it can also inhibit termination. In general, the results are exciting however the manuscript suffers from its brevity. Furthermore, the data for the main conclusion of the paper that PF846 has a unique mechanism of action to allow termination to occur but trap the NC on the ribosome after hydrolysis, does not in my opinion, fully support the conclusion. I believe that it inhibits termination, but I am not convinced that it is after peptide bond formation based on the presented data.

We thank the reviewer for this critique, which we addressed in our response to reviewer 1, above.

Major comments

1. Fig 2. It needs to be made clear whether the cryo-EM map densities are segmented or not. I would presume that they segmented for the colored ones and not for the inset. However, this needs to be always stated clearly. Moreover, different thresholds need to be shown for the unsegmented map to see if density does in fact connect the A76 with the NC at lower thresholds. Either way I don't find this evidence to make the statement on page 6 that it indicates hydrolysis of the ester bond without supporting biochemistry, which has not been performed up to this point in the paper.

Thank you for the suggestions. We clarified this in the figure legend of **Fig. 2** in the revised manuscript: "Surface representations of the segmented cryo-EM density for eRF1, P-site tRNA, PF846, and ribosomal proteins are indicated." We have moved the inset into **ED Fig. 7b**. For **ED Fig. 7b**, we used a single threshold for the different ligands in visualizing the density map to get a correct sense in the gap of the density. We now note this in the figure legend.

As described in the main response above, we now have biochemical evidence that the CDH1-NPN NC is trapped in the ribosome exit tunnel after release from P-site tRNA in a PF846-independent manner. In the conditions of our cryo-EM sample preparation, both NC-tRNA and released NC are present in the sample (See updated **ED Fig. 7c**). Consistent with this, when we lower the contour of the cryo-EM map, we observe a connection in the density between the NC and P-site tRNA representing the ester bond. We have added a panel to **ED Fig. 7** showing this (**ED Fig. 7b**).

2. In Ed Fig. 6, the authors show that the NC-tRNA becomes "slowly hydrolyzed on the hour timescale in RNCS purified from in vitro translation reactions" – the authors need to add the important point "in the presence of PF846". The RNC is rapidly hydrolyzed even on ice in the absence of the drug! More importantly however I fail to understand how these findings fit with their results…one would presume that isolation of an RNC for cryo-EM studies is not done at room temperature but on ice or 4deg, Moreover, it would

seem most relevant to actually perform the experiments on the sample that was used for the cryo-EM study (or at least treated the same way) to see if the NC-tRNA is intact?

Thank you for pointing this out, we reworded the whole paragraph in the revised manuscript to take into account our new model, as described above. In fact, the RNC sample isolated for cryo-EM was done at room temperature, to reduce nonspecific binding of 80S particles to the anti-FLAG beads, as described in the methods: "The supernatant was incubated with 50 μL of anti-FLAG M2 agarose beads (Sigma, A2220) for 1 hour at room temperature with gentle mixing. All the following purification steps were conducted at room temperature to avoid nonspecific binding of 80S particles to the beads unless specifically noted." Based on the original and new experiments in **ED Fig. 7** and **8**, we think the cryo-EM sample had a mixture of RNCs with NC-tRNA and terminated NC. As noted above, weak density is present between the NC and P-site tRNA, consistent with this interpretation.

3. I do not see the pivotal experiment showing that the NC remains on the ribosome dependent on the presence of the drug. This could be done by monitoring association of the NC with the ribosome fraction after pelleting assays, in presence and absence of the drug. Controls would include puromycin to show that the NC is released when the drug is not present and when the drug is added, puromycin should still lead to loss of the peptidyl-tRNA band, but the NC should still be detected in the ribosome fraction in a drug-dependent fashion. In fact, dose-dependence of the drug would be the most convincing.

We thank the reviewer for the suggestions. We have updated our model based on the new data added as described in the overall response, which is consistent with the reviewer's model, namely that PF846 inhibits eRF1-dependent hydrolysis of the NC from P-site tRNA.

4. The authors discuss their results within the context of termination and seem to have forgotten that there is an elongation effect, for example, for ED Fig. 1 and ED Fig. 6, it seems inappropriate to state here inhibition of translation termination since this assay does not monitor specifically termination. In fact, the structural analysis shows that it's a mixture of inhibition of termination and elongation complexes. How does this influence the interpretation of the other results in the paper e.g. Fig. 3e and ED Fig. 8-9. Might one argue based on particle numbers from ED Fig. 2 that termination if 66% and elongation is 33% of the complex?

We thank the reviewer for this comment, and think the text was unclear. We agree, and have updated our model in the main text as described in our overall response above.

5. Lastly, assuming that the authors are correct and that the inhibition by PF846 is post-hydrolysis of the NC by eRF1, then it is unclear to me which step is then inhibited? I-i.e. is it eRF1 dissociation? How does PF846 prevent eRF1 dissociation then? A simpler model would be that eRF1 remains because it cannot hydrolyze the ester linker.

Please see our overall response above, which updates the model for PF846 action.

Minor comments

1. The manuscript suffers from its brevity, presumably having fallen down from being sent to another journal with tighter space restrictions. For example, the entire paper is based on observations that PF846 inhibits not only elongation but also termination as reported in reference 4 but no details are given. Maybe the evidence should be summerized in the introduction too? Why were sequences NPN* added to Cadherin-1? The rationale for the initial results is lacking in this manuscript with the expectation that the reader should first read reference 4. Is it at a particular stop codon? How did it come about that GCV is the selected control sequence in reference 4?

Thank you for the comments. We now describe the basis for the present manuscript more clearly in the beginning of the Results section. We have summarized more information about the previous study of PF846 in the revised manuscript, so that readers will have an easier time understanding the results of the present study.

Moreover, the discussion is incomplete with reference to other stalling mechanisms and surprisingly, does not even discuss the numerous studies reporting antimicrobial peptides that block termination after the release factor has hydrolyzed the ester linkage.

In the revised manuscript, we added the antimicrobial peptides in the discussion and cited relative references (Florin et al. 2017; Mangano et al. 2020; Graf et al. 2018). “For example, the recently described bacterial termination-specific inhibitor apidaecin (Api), a 19 amino acid long proline-rich antimicrobial peptide, binds to the ribosome exit tunnel when no NC is present and traps the release factors (RF1 or RF2) in the A-site by stable interactions with its C-terminal amino acid [44–46]. By contrast, PF846-stalled termination suppresses translation termination prior to eRF1-directed hydrolysis of the NC-tRNA ester bond.”

2. Page 3: There lacks clarity in the last sentence of the first paragraph. Treatment of cells with PF846 inhibits luciferase fused to CDH-NPN*…this is seen in Fig. 1a however because it’s a log scale without many points on the x-axis its hard to understand how the 3uM IC50 was calculated.

Thank you for the comments. In the revised manuscript, we added the concentrations of PF846 in the figure legends: “(**a**) Luciferase reporter assays of CDH1–NPN* (black circles) and CDH1–GCV* (magenta circles) stable cell lines in response to PF846 with different concentrations of 0, 1, 3, 5, 7 10, 20, 50 and 100 μM”. To better see the IC50, we added a vertical line (revised **Fig. 1a** and **ED Fig. 10h**).

That CDH1-GCV is not inhibited is not true, there is also inhibition seen in Fig. 1a, just not as dramatic.

PF846 barely affects CDH1-GCV* in cells unless using very high concentrations such as 50 μM and 100 μM, and never reaches 50% inhibition in our experiments. We reworded the description for CDH1-GCV* in the revised manuscript: "Treatment of cells with PF846 showed that the compound inhibits the expression of nanoluciferase fused to CDH1-NPN*, with an IC50 around 4 μM, but only weakly inhibits expression of nanoluciferase fused to CDH1-GCV* at high doses of PF846 (revised **Fig. 1a and Extended Data Fig. 1**)." Also, based on our new *in vitro* experiments (**ED Fig. 7** and **8**), CDH1-GCV* is not inhibited by PF846 in terms of NC release from P-site tRNA, even at 50 μM. It is possible that in cells, high concentrations of PF846 are able to trap the released NC in the ribosome exit tunnel, but we do not want to speculate as to the mechanism based on the data we have.

To help the reader, perhaps the authors should consider putting the (Fig. 1a) at the end of this sentence rather after the "consistent with in vitro translation results". One assumes then that the in vitro is ED Fig. 1...actually Fig. 1b…but this also seems to be an "in cell assay" as indicated by the title. So if Fig. 1b is also in cells, where is the in vitro data? Maybe the authors expect the reader to understand that this is in reference 4? Also with regard to ED Fig. 1b, the small symbols in the key of the figure cannot be seen clearly…perhaps just use black and white squares as indicated in the legend?

Thank you for the suggestions. We have reworded the Introduction and clarified the results to more clearly indicate what is going on in **Figure 1** and **ED Figure 1**.

3. On page 6 (para 2, line 9-10), I find it inappropriate to cite Fig. 2a but rather only ED Fig. 6 at this point. Especially since I don't find anything in the biochemistry consistent with the break in the density…see major points above.

We have rewritten that entire paragraph in light of our new data and updated model.

4. The use of PCSK9 is confusing. The authors write that it is "unlikely to form a compact a-helical geometry" but then show Phyre predictions of a-helices? It's also unclear why the authors now change the format of the presentation to treat all samples with RNase A and do not use the representation as in Fig. 3e. Also using a positive and negative control would be good.

For the PCSK9 stalling sequence, Phyre2 predicts that the sequence would not adopt a continuous α-helix as we observe with CDH1-NPN*. Rather the helical segments are interrupted by predicted unstructured regions. To make the experiments more clear, we have combined previous **ED Fig. 6** and **ED Fig. 8**, since we have now used a PCSK9-NPN construct PN35* (no RNase A treatment) in our kinetics experiments. Notably this sequence is somewhat resistant to NC-tRNA cleavage by eRF1 even in the absence of PF846, and release is only slightly inhibited in the presence of the drug. Consistent with weak inhibition, the NC-tRNA disappears nearly completely with time (**ED Fig. 8b**). We also added all the RNase A-treated CDH1-NPN* mutation data in **ED Fig. 10f-g**.

5. In Fig. 3e, it is unfortunate that every mutation basically abolishes the stalling. The reader has to look to ED Fig. 9 to be convinced that there are amino acid position specific effects. Perhaps it would be good to have a few negative control mutations in Fig. 3e too.

Thank you for the suggestion. We have reorganized **Fig. 3e** and **ED Fig. 9** (new **ED. Fig 10**), based on these comments.

6. If an intact NPN motif is needed, how many proteins have intact NPN before a stop codon in the human genome? Obviously, more than an NPN motif is needed, so how many of these NPN* proteins are affected by PF846? Are there proteins also affected during elongation too? i.e. are these activities separable? Is there a secondary structure feature requirement before the NPN motif? Do mutations that break the helix also lead to loss of stalling?

These are interesting questions, and have helped us in revising the manuscript for clarity. Ribosome profiling data identified only 18 nascent chain sequences that can be stalled by PF846 during translation elongation (Lintner *et al.*, 2017, *Plos Biology*). With such little sequence information, we could not identify a simple amino acid motif or structural property responsible for PF846-dependent stalling. To overcome this paucity of data, we performed an mRNA library experiment by randomizing 4 amino acids in the CDH1 stalling sequence near the PTC to identify the sequence preference for PF846-mediated translation stalling (Li *et al*., 2019*, NSMB*). For example, we identified an HYHS sequence motif near the PTC. However, this sequence motif is not sufficient to induce PF846-dependent stalling but requires additional sequences in the nascent chain (Li *et al*., 2019*, NSMB*).

We also identified different sequence motifs near the PTC adjacent to the stop codon, exemplified by NPN*, that led to PF846-dependent inhibition of translation termination. By ribosome profiling, we did not have the ability to detect whether PF846 inhibited translation termination (Lintner *et al.*, 2017, *PLoS Biology*). Searching the human genome, when we use the motif pattern [DNHY][PV][A-X], we identify 447 total proteins encoding this motif by the stop codon, including one example of NPN*. We have added this information to the revised manuscript. The loss of activity seen with PCSK9

termination constructs and the mutagenesis experiments in the CDH1-NPN context indicate that NC sequence and secondary structure beyond the NPN* motif will be necessary to induce inhibition of termination (new **ED Fig. 8**). Given that the sequence requirements for PF846-dependent inhibition of translation termination extend beyond this motif, it will be interesting to examine whether any of these proteins are affected by PF846 in the future.

Reviewer #1 (Remarks to the Author):

The revised paper is good, important and convincing.

Reviewer #2 (Remarks to the Author):

The authors have addressed my comments and in particular my main concern by performing the necessary experiment to show that their initial model was incorrect and in fact the drug does not trap the nascent chain on the ribosome after release but rather slows down peptide-tRNA hydrolysis. I am a little surprised that given this experiment is now describing the mechanism of action that this in not in the main figures but rather in the supplementary. The authors might reconsider this?

Maybe the authors wants to also consider toning-down some of their language, especially in the abstract where they claim "this unprecedented mechanism of action"...there is nothing unprecedented about it...there are many examples of drugs that work with the NC to prevent peptide-tRNA hydrolysis. The authors might argue but these are during elongation but actually for ErmCL for example the A-site can be a stop codon, therefore the inhibition is also during termination. If the authors really want to persist with using the term unprecedented then they will need to add another qualifier "targeting eukaryotic translation"...but then they could also explain to me what the "new principles" are that they revealed for translation termination? Alternatively they could just write a less flowery final sentence?

Reviewer #1 (Remarks to the Author):

The revised paper is good, important and convincing.

We thank the reviewer for finding our manuscript interesting.

Reviewer #2 (Remarks to the Author):

The authors have addressed my comments and in particular my main concern by performing the necessary experiment to show that their initial model was incorrect and in fact the drug does not trap the nascent chain on the ribosome after release but rather slows down peptide-tRNA hydrolysis. I am a little surprised that given this experiment is now describing the mechanism of action that this is not in the main figures but rather in the supplementary. The authors might reconsider this?

Maybe the authors want to also consider toning-down some of their language, especially in the abstract where they claim "this unprecedented mechanism of action"...there is nothing unprecedented about it...there are many examples of drugs that work with the NC to prevent peptide-tRNA hydrolysis. The authors might argue but these are during elongation but actually for ErmCL for example the A-site can be a stop codon, therefore the inhibition is also during termination. If the authors really want to persist with using the term unprecedented then they will need to add another qualifier "targeting eukaryotic translation"...but then they could also explain to me what the "new principles" are that they revealed for translation termination? Alternatively they could just write a less flowery final sentence?

We thank the reviewer for the positive comments and suggestions.

We have moved previous **Supplementary Fig. 8c** to **Fig. 2c** since it is helpful in explaining our model.

PF846 is the first identified small molecule that targets eukaryotic translation termination. For ErmCL-stalled translation noted by the reviewer, there is no evidence showing that the macrolide erythromycin can stall translation termination. From the structures of ErmCL-stalled ribosomes, access to the A site is blocked due to the distinct conformation of A2602 (Arenz et al. 2016; Arenz et al. 2014) as we show in **Fig. 5c**. When we superimpose our structure on these models, the GGQ hairpin of eRF1 would collide with A2602. Thus, release factors cannot bind to the ribosome in macrolide-dependent stalling mediated by ErmCL.

To satisfy the concerns of the reviewer, we first added “eukaryotic” in the abstract. We cite the structural studies for ErmCL and clarify that the A site is blocked for tRNA and release factor binding when discussing the macrolide-dependent stalling mechanism: “However, in contrast with macrolide-dependent stalling in bacteria, PF846 does not lead to nucleotide rearrangements that would block tRNA and release factor access to the A site in the PTC (Arenz et al. 2016; Arenz et al. 2014) (**Fig. 5c-d**).”
